# Supplementary material for: HIT-MAP: A scalable approach to multimodal mapping of subcellular organization
Source: Cell Rep Methods. 2026 Jul 22;6(9):101534. doi: 10.1016/j.crmeth.2026.101534 (PMC13615500; doi:10.1016/j.crmeth.2026.101534)
Supplement: Supplementary file 1 [file mmc1.pdf]

Cell Reports Methods, Volume *6*

Supplemental information

HIT-MAP: A scalable approach to multimodal mapping of subcellular organization

Gege Qian, Joonwon Kim, Richa Tiwari, Benjamin J. Polacco, Antoine Forget, Leah V. Schaffer, Hark Kyun Kim, Jiahao Gao, Yuan Zhou, Gwendolyn M. Jang, Marcus R. Kelly, Xiaoyu Zhao, Helene Foussard, Liudeng Zhang, Nevan Krogan, Trey Ideker, and Alejandro Chavez

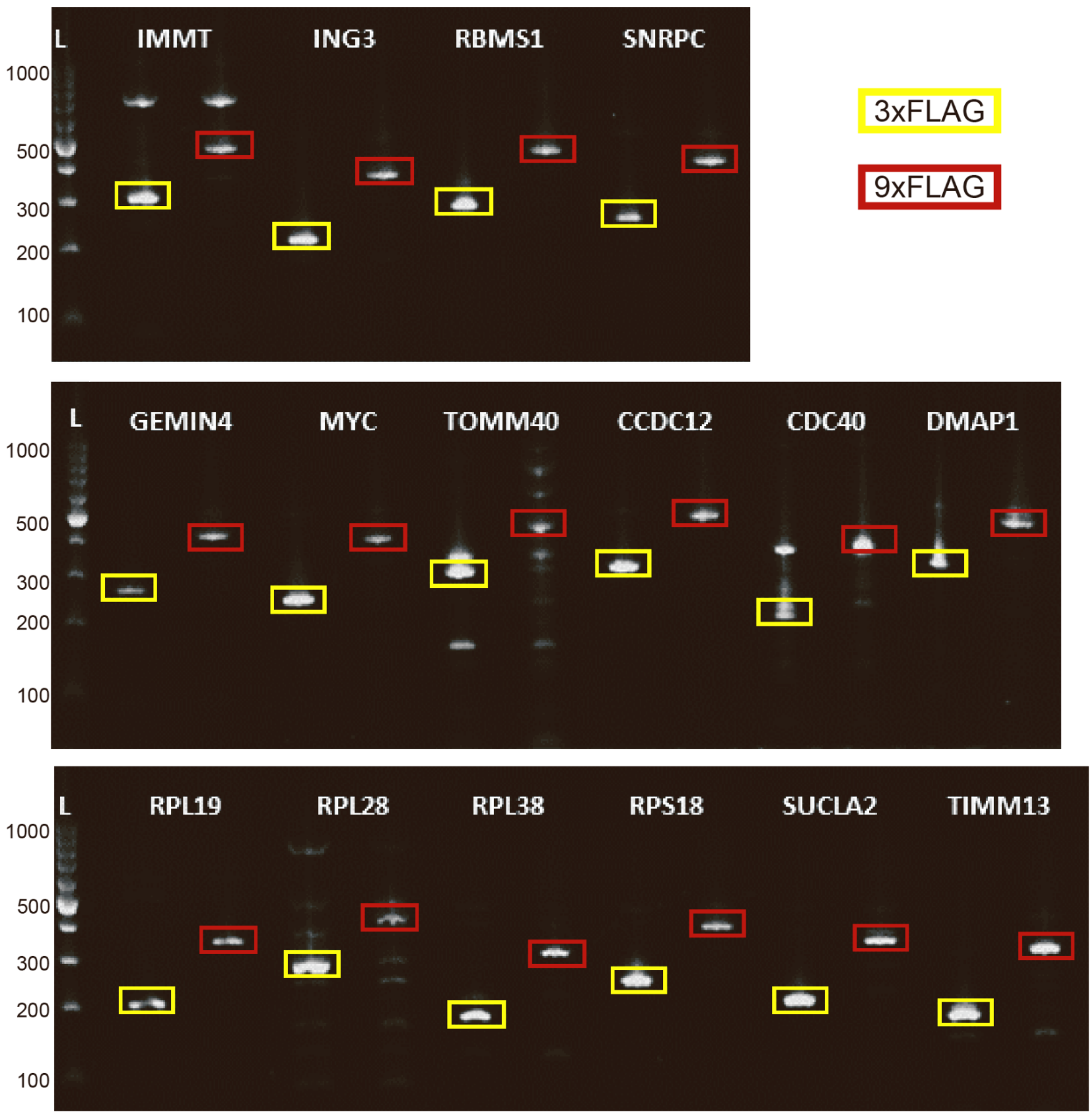

Figure S1. PCR validation for all tagged cell lines.

Knock-in is validated using PCR with one forward primer binding to the target gene and the reverse primer binding to the T2A sequence downstream of the FLAG tag. This approach ensures that PCR products are generated only when the tag is inserted in the correct orientation at the C-terminus of each target gene. The 9×FLAG construct shows larger PCR products due to the additional 6 FLAG repeats compared to the single FLAG tag.

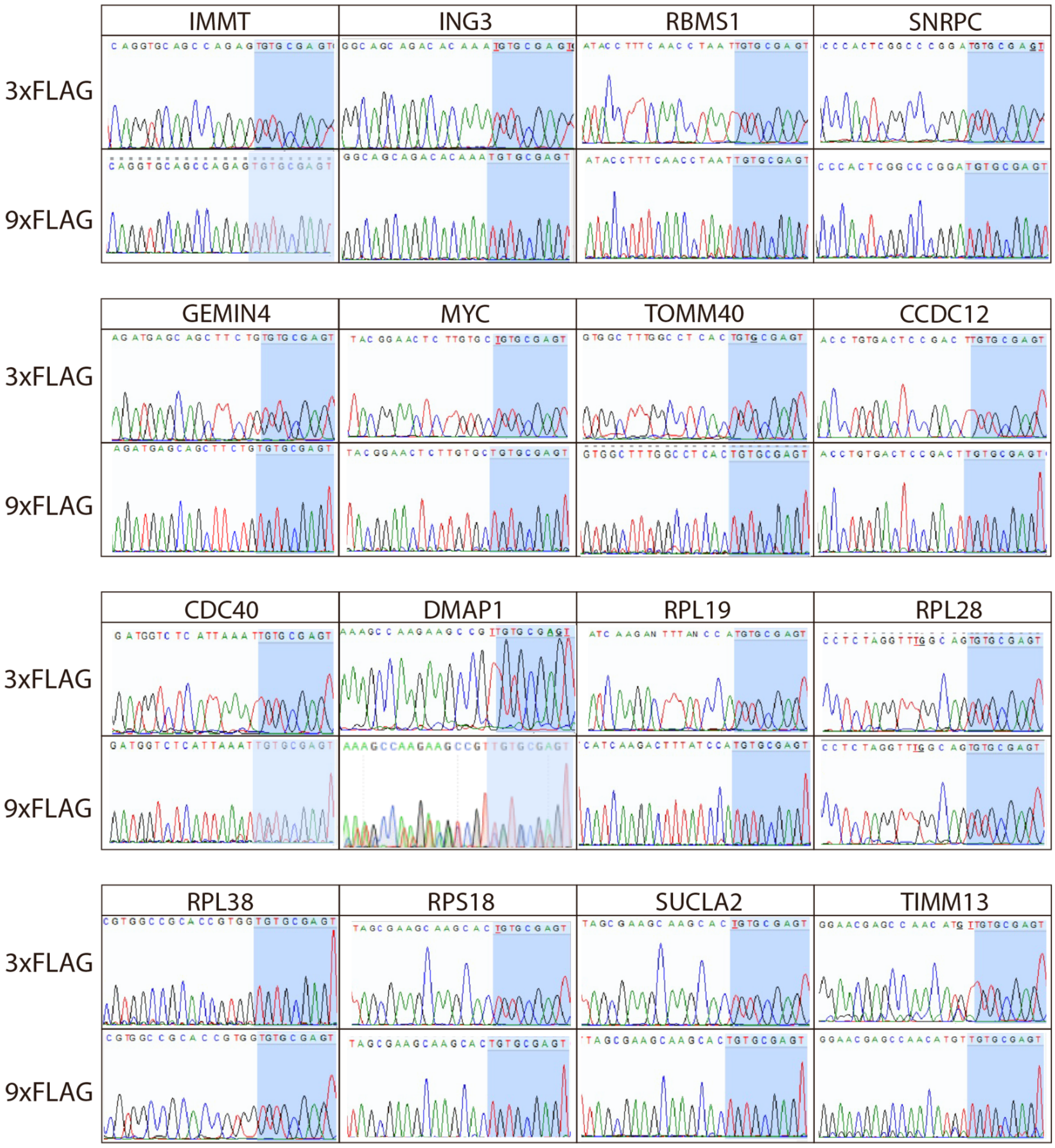

Figure S2. Sanger sequencing validation.
Sequencing analysis of PCR products confirms successful tag integration at the C-terminus of each gene. The target gene sequence (5' to 3') appears on the left, and the blue-colored section indicates where the linker sequence begins.

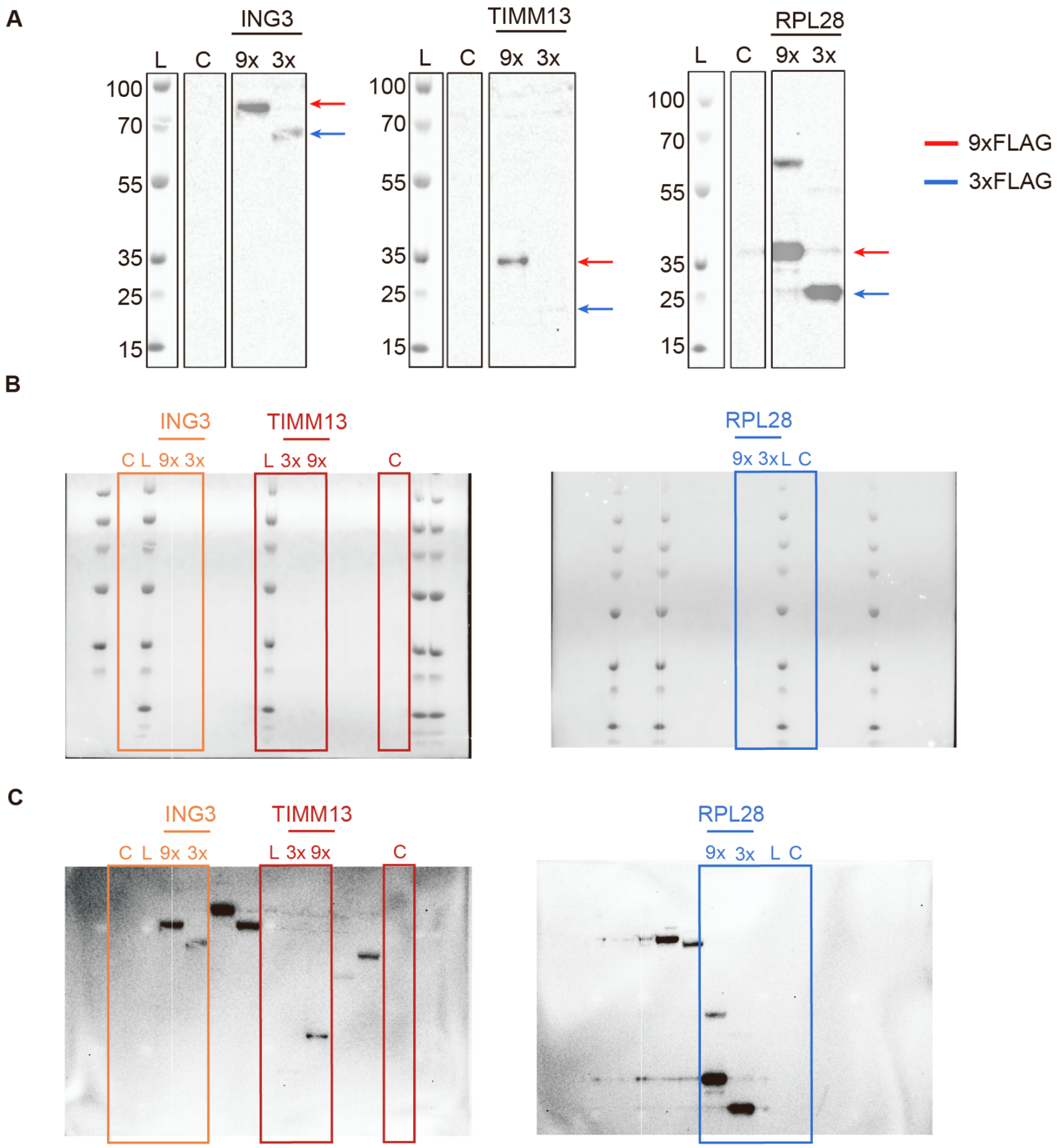

Figure S3. Western blot validation of tagging analysis.

a) Western blot analysis demonstrating the fusion of either a 9xFLAG- or 3xFLAG-tag to ING3, TIMM13, and RPL28. Red arrows indicate the 9xFLAG-tagged variant of the proteins and the black arrows indicate the 3xFLAG-tagged versions. Proteins are detected using an anti-FLAG antibody. The 9xFLAG-tagged proteins consistently showed stronger signal detection and higher molecular weight compared to their 3xFLAG-tagged counterparts (9xFLAG tag 10kDa, 3xFLAG tag 4kDa). L: ladder. C: Control untagged sample. 9x: 9xFLAG-tagged protein samples. 3x: 3xFLAG-tagged protein samples. Original image of western blot with b) colorimetric and c) chemiluminescence detection. The images are edited using imageJ to remove background noise and adjusted uniformly along all the images for clarity.

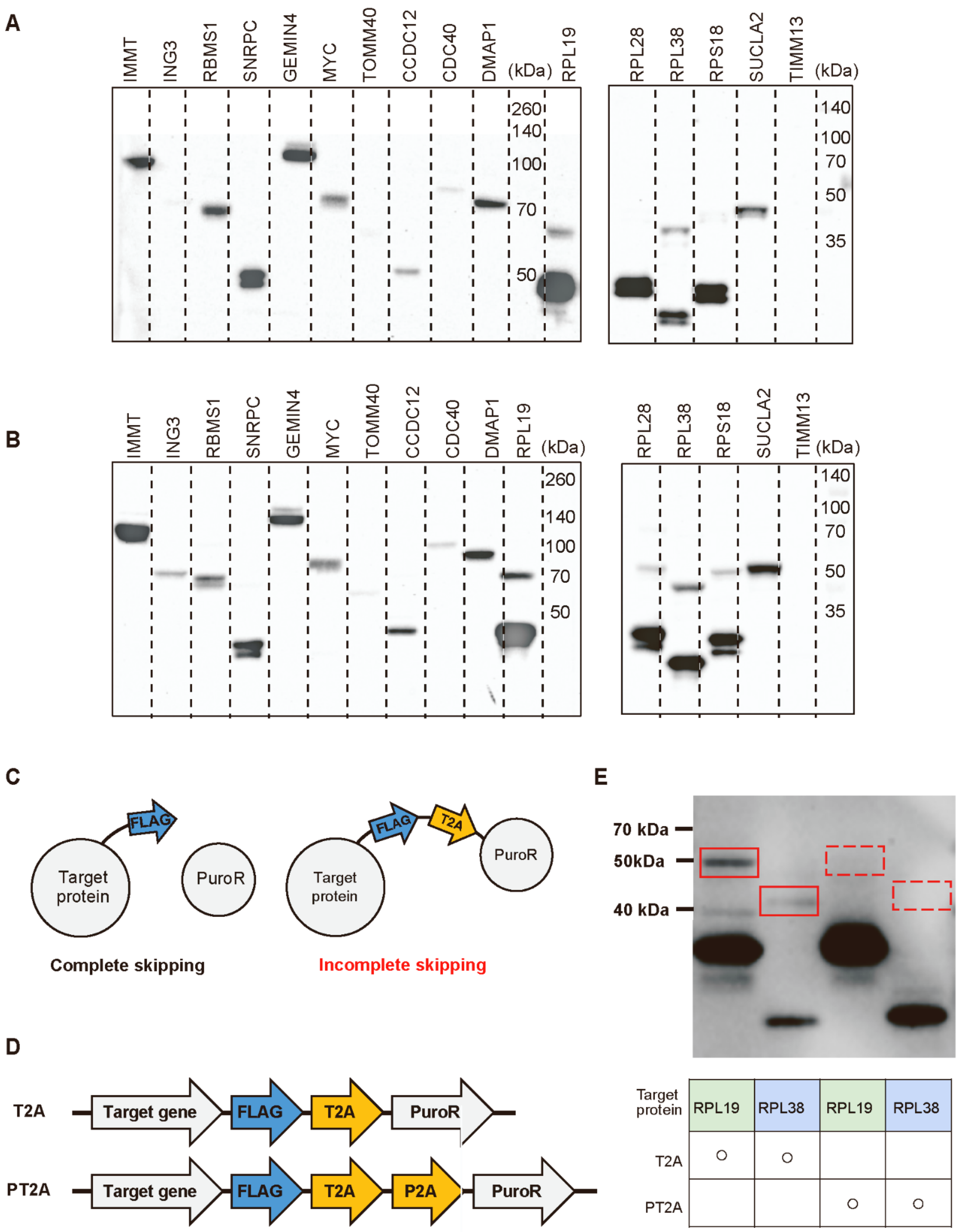

Figure S4. Western blot validation for all tagged cell lines.
Western blot validation of a) 3xFLAG-tagged cell lines and b) 9xFLAG-tagged cell lines. Lane labels indicate the name of the tagged gene in each lane. Both blots are generated using equal protein input amounts and equivalent exposure times. c) Higher molecular weight bands show in 9xFLAG-tagged proteins (e.g. RPL28) represent minor populations of uncleaved T2A-PuroR fusion products, which are more detectable due to enhanced antibody binding to the 9xFLAG tag compared to that of 3xFLAG tag. d,e) Further optimization using tandem skipping peptides (PT2A) effectively resolves incomplete cleavage, eliminating the high molecular weight fusion bands.

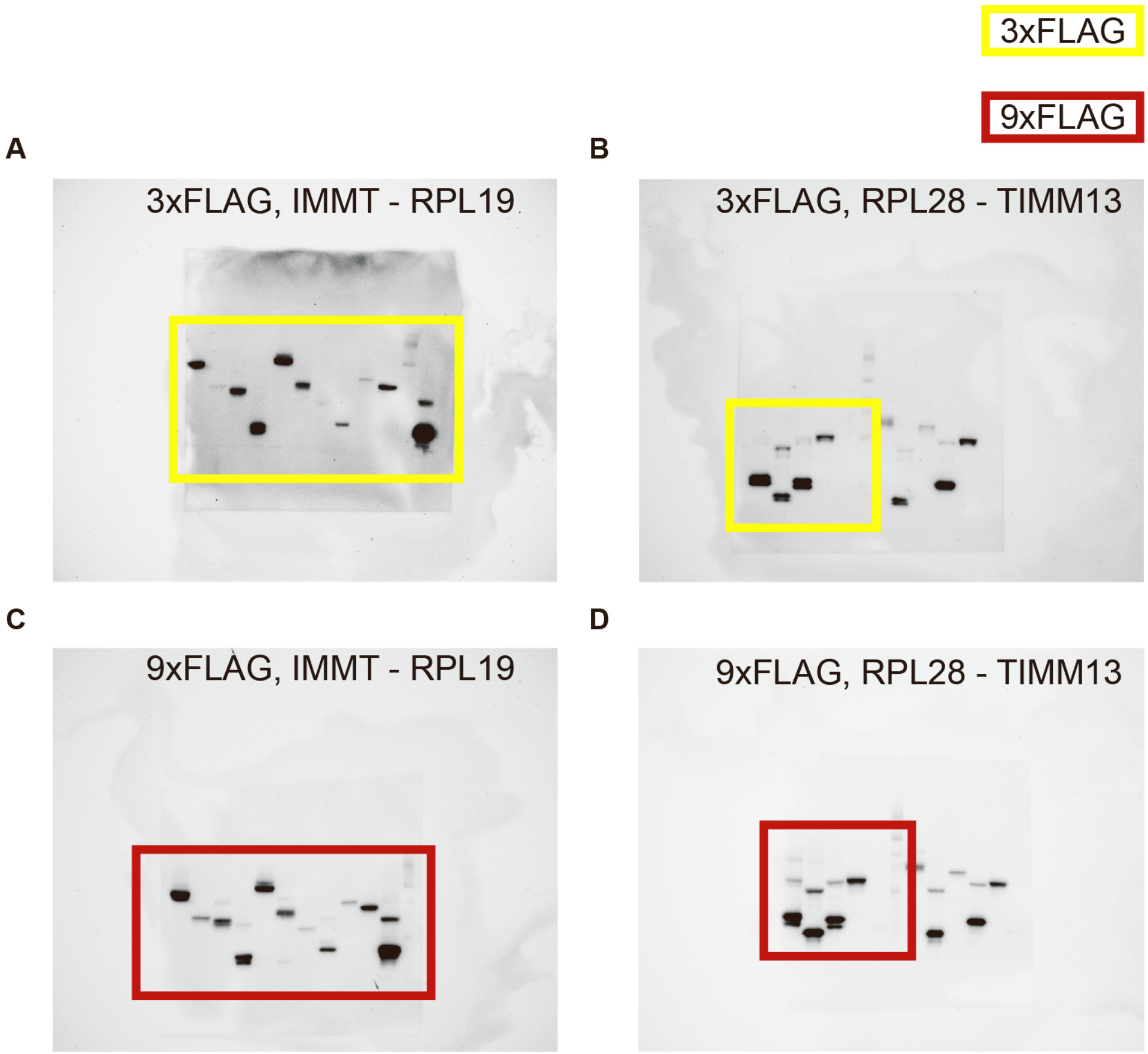

Figure S5. Raw image of Western blot validation for all tagged cell lines. a-b) 3xFLAG-tagged cell lines and c-d) 9xFLAG-tagged cell lines. The area inside the box indicates the region that has been presented in Supplemental Figure 4.

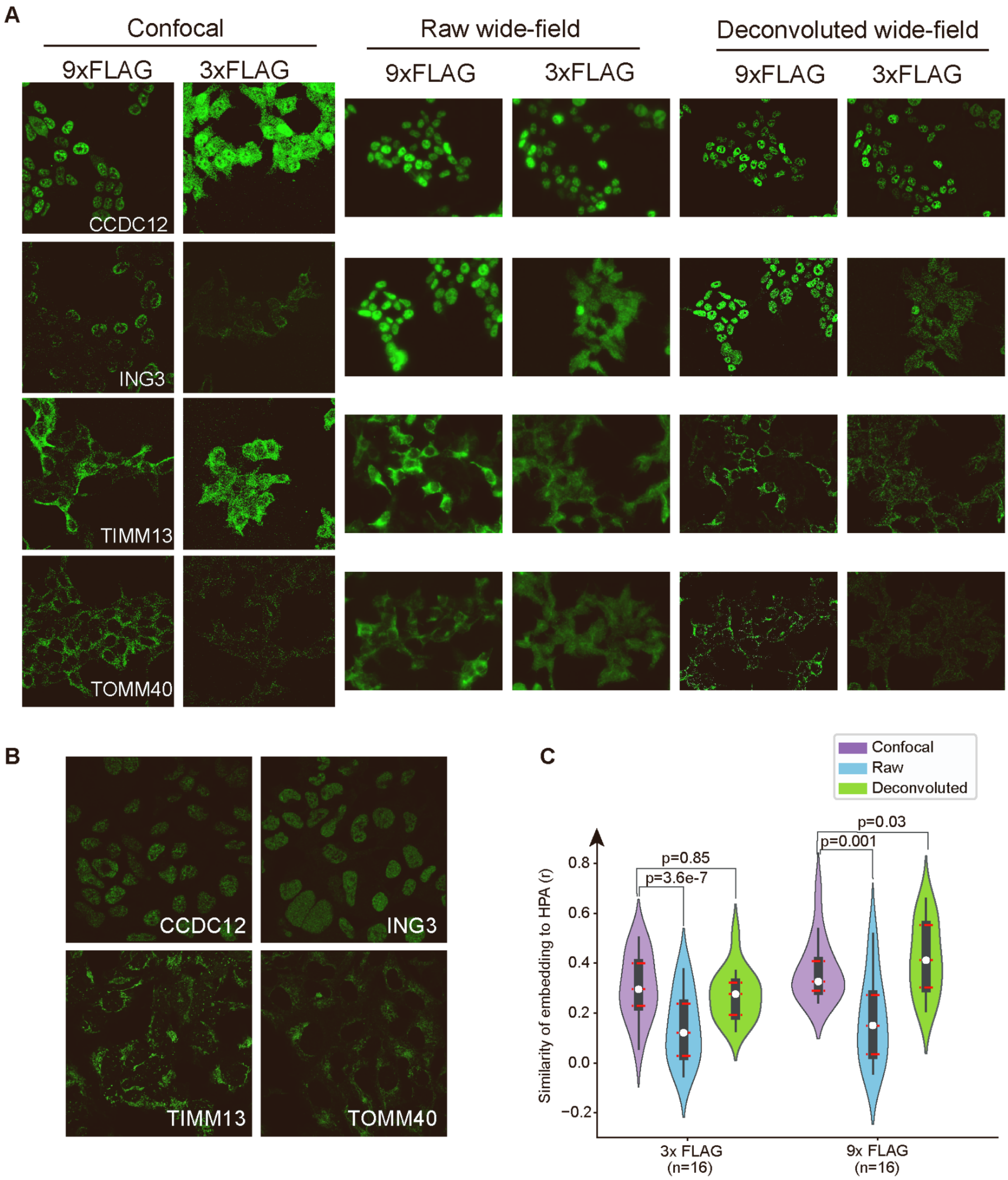

Figure S6. Tagging system validation with in-house confocal images

a) Example confocal, raw wide-field and deconvolved wide-field images of 3xFLAG and 9xFLAG tagged cell lines of CCDC12, ING3, TIMM13 and TOMM40. b) Confocal image from HPA.

c) Distribution of Pearson correlations between embeddings of different imaging platforms and image processing approaches to that of HPA for all 16 proteins tagged with either 3xFLAG or 9xFLAG.

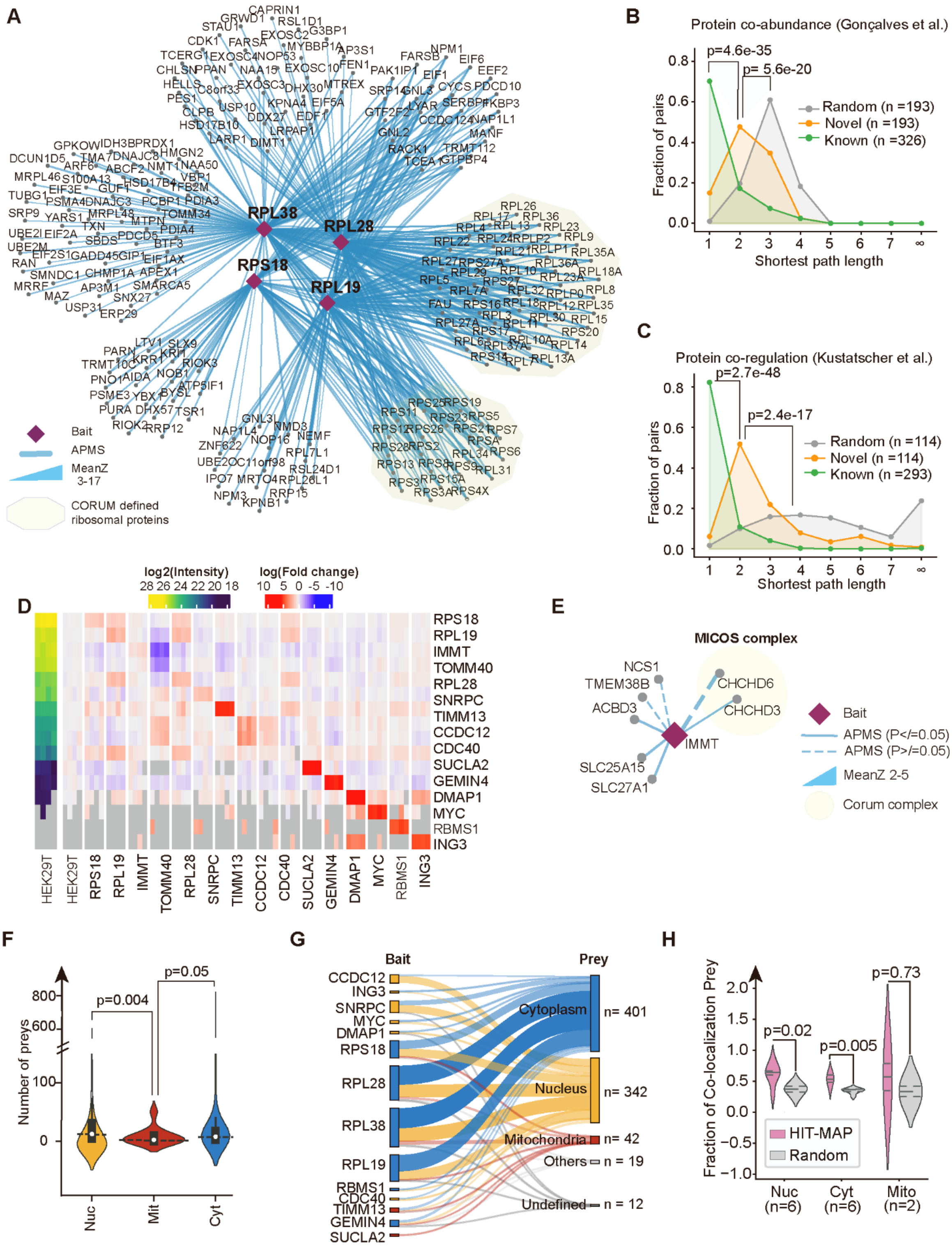
A
RPL38
RPL28
RPS18
RPL19
Bait
APMS
MeanZ
3-17
CORUM defined
ribosomal proteins
B
Protein co-abundance (Gonçalves et al.)
p=4.6e-35
p= 5.6e-20
Random (n =193)
Novel (n =193)
Known (n =326)
Fraction of pairs
Shortest path length
C
Protein co-regulation (Kustatscher et al.)
p=2.7e-48
p=2.4e-17
Random (n =114)
Novel (n =114)
Known (n =293)
Fraction of pairs
Shortest path length
D
log2(Intensity)
28 26 24 22 20 18
log(Fold change)
10 5 0 -5 -10
RPS18
RPL19
IMMT
TOMM40
RPL28
SNRPC
TIMM13
CCDC12
CDC40
SUCLA2
GEMIN4
DMAP1
MYC
RBMS1
ING3
HEK29T
HEK29T
E
MICOS complex
NCS1
TMEM38B
ACBD3
CHCHD6
CHCHD3
IMMT
SLC25A15
SLC27A1
Bait
APMS (P</=0.05)
APMS (P>/=0.05)
MeanZ 2-5
Corum complex
F
Number of preys
p=0.004
p=0.05
Nuc
Mit
Cyt
G
Bait
Prey
CCDC12
ING3
SNRPC
MYC
DMAP1
RPS18
RPL28
RPL38
RPL19
RBMS1
CDC40
TIMM13
GEMIN4
SUCLA2
Cytoplasm
n= 401
Nucleus
n= 342
Mitochondria
n= 42
Others
n = 19
Undefined
n = 12
H
Fraction of Co-localization Prey
p=0.02
p=0.005
p=0.73
HIT-MAP
Random
Nuc
(n=6)
Cyt
(n=6)
Mito
(n=2)

Figure S7. AP-MS PPI analysis.

a) AP-MS detected PPI full network for ribosomal proteins, highlighting CORUM complex defined ribosomal preys. For PPI mapping, a cut-off of Mean $Z_{MADEX} \geq 3$ and $p \leq 0.01$ was used. b) Distribution of the shortest distances of the PPIs identified from AP-MS grouped into Known (included in STRING and CORUM), Novel (not documented) and Random selected set of proteins in the high confidence graph generated from protein co-abundance data (Goncalves et al.) and c) protein co-regulation data (Kustatscher et al.) d) Heatmap of mass-spectrometry intensity for all baits. The left most column shows the intensity of the negative control of HEK293T cells with no protein tagged. Red/blue heatmap shows the log2fold change of each bait compared to HEK293T control. Note: RBMS1 and ING3 were not detected in the negative control. Their fold change is their intensity divided by the intensity of MYC in the HEK293T control. e) AP-MS detected PPI network for IMMT with a relaxed threshold ($Z_{MADEX} \geq 2$). CORUM defined preys are highlighted. f) Violin plot showing the number of preys for baits annotated by their subcellular localization in OpenCell dataset (https://opencell.sf.czbiohub.org/download). Asterisks(*) indicate significantly more preys than the mean of mitochondrial baits ($p < 0.05$). g) Sankey plot with the flow representing the interaction of a bait with preys assigned to specific compartments, illustrating the distribution of interaction partners across different cellular localizations. h) Violin plot showing the percentage of prey proteins localized to the same subcellular compartment as their corresponding bait. Distributions are compared to a randomized control in which prey are randomly assigned to baits. Statistical significance was assessed using pairwise Student's t-tests, and p-values are indicated.

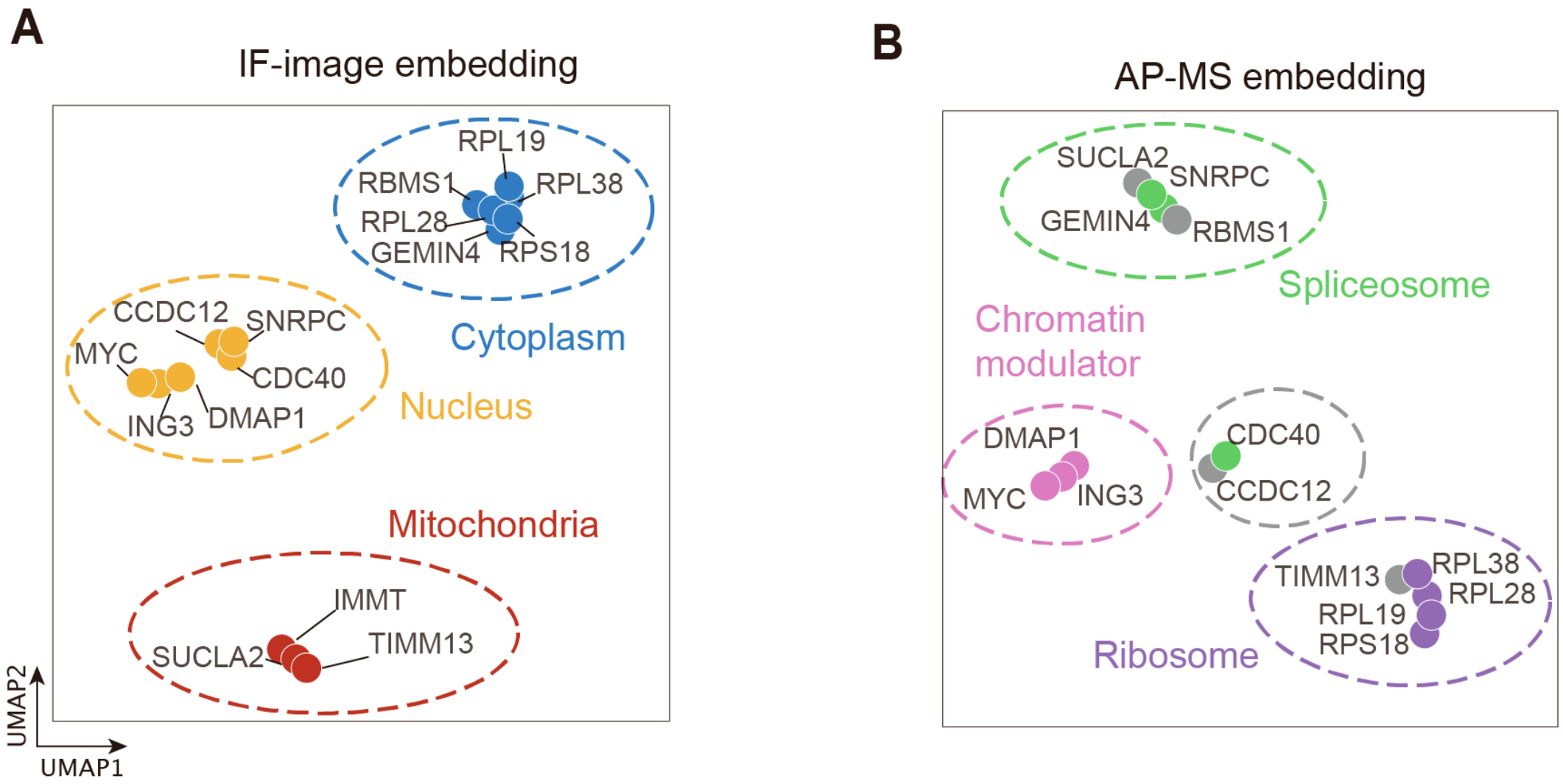

Figure S8. UMAP plot of IF and AP-MS embedding.
a) IF embedding UMAP clusters are annotated with their known subcellular localizations. b) AP-MS embedding UMAP clusters are labeled based on the members within a known complex, the gray dots mean the protein is not known to be part of the complex annotated.

A

Sample #	Tagged cell generation				
	Plasmid library	gRNA cell library	Tagged cell libraries	Clonal cell isolation and validation	Total
100	662	238	336	2,976	4,213
500	2,112	249	387	12,810	15,558
1,000	2,309	272	738	25,404	28,723
5,000	3,336	546	3,690	126,362	133,934
10,000	4,321	890	7,380	252,524	265,115

B

Sample #	Conventional method		HIT-MAP		
	Direct antibody	Total	Tagged cell generation	Anti-tag antibody	Total
100	30,000	30,000	4,213	300	4,513
500	150,000	150,000	15,558	1,200	17,058
1,000	300,000	300,000	28,723	2,400	31,723
5,000	1,500,000	1,500,000	133,934	12,000	148,934
10,000	3,000,000	3,000,000	265,115	24,000	295,115

C

Sample #	Conventional method				HIT-MAP		
	CDS synthesis	Cloning and lentivirus production	Cell culture	Total	Tagged cell generation	Cell culture	Total
100	10,800	5,200	1,575	17,575	4,213	6,300	10,513
500	54,000	26,000	7,875	87,875	15,558	31,500	47,058
1,000	108,000	52,000	15,750	175,750	28,723	63,000	91,723
5,000	540,000	260,000	78,750	878,750	133,934	315,000	448,934
10,000	1,080,000	520,000	157,500	1,757,500	265,115	630,000	895,115

Figure S9. Cost analysis of HITAG-based clonal cell line generation and downstream HIT-MAP applications.

a) Cost breakdown for generating isolated clonal cell lines using HITAG, including gRNA plasmid library construction, integration of gRNAs into cells, tagged cell library construction, and clonal cell isolation and validation. b, c) Cost comparison between conventional methods and HIT-MAP for imaging and AP-MS. For fair comparison, HIT-MAP costs include the tagged cell generation expense, which can be shared across multiple applications for greater cost efficiency but is added at full cost to both IF and AP-MS tallies listed above in case future groups are only interested in using the tagged lines for a single experimental output. The cost comparison evaluates material costs that differ between HIT-MAP and conventional methods during sample preparation and experimental execution. The conventional method costs for imaging were calculated based on the target-specific primary antibodies while AP-MS costs included CDS synthesis, cloning, and transfection/transduction. Detailed costs and parameters are provided in Supplemental Table 16-18. All costs are shown in USD.
