# Supplementary material for: HIT-MAP: A scalable approach to multimodal mapping of subcellular organization
Source: Cell Rep Methods. 2026 Jul 22;6(9):101534. doi: 10.1016/j.crmeth.2026.101534 (PMC13615500; doi:10.1016/j.crmeth.2026.101534)
Supplement: Supplementary file 2 [file mmc22.pdf]

Cell Reports Methods

Article

HIT-MAP: A scalable approach to multimodal mapping of subcellular organization

Graphical abstract

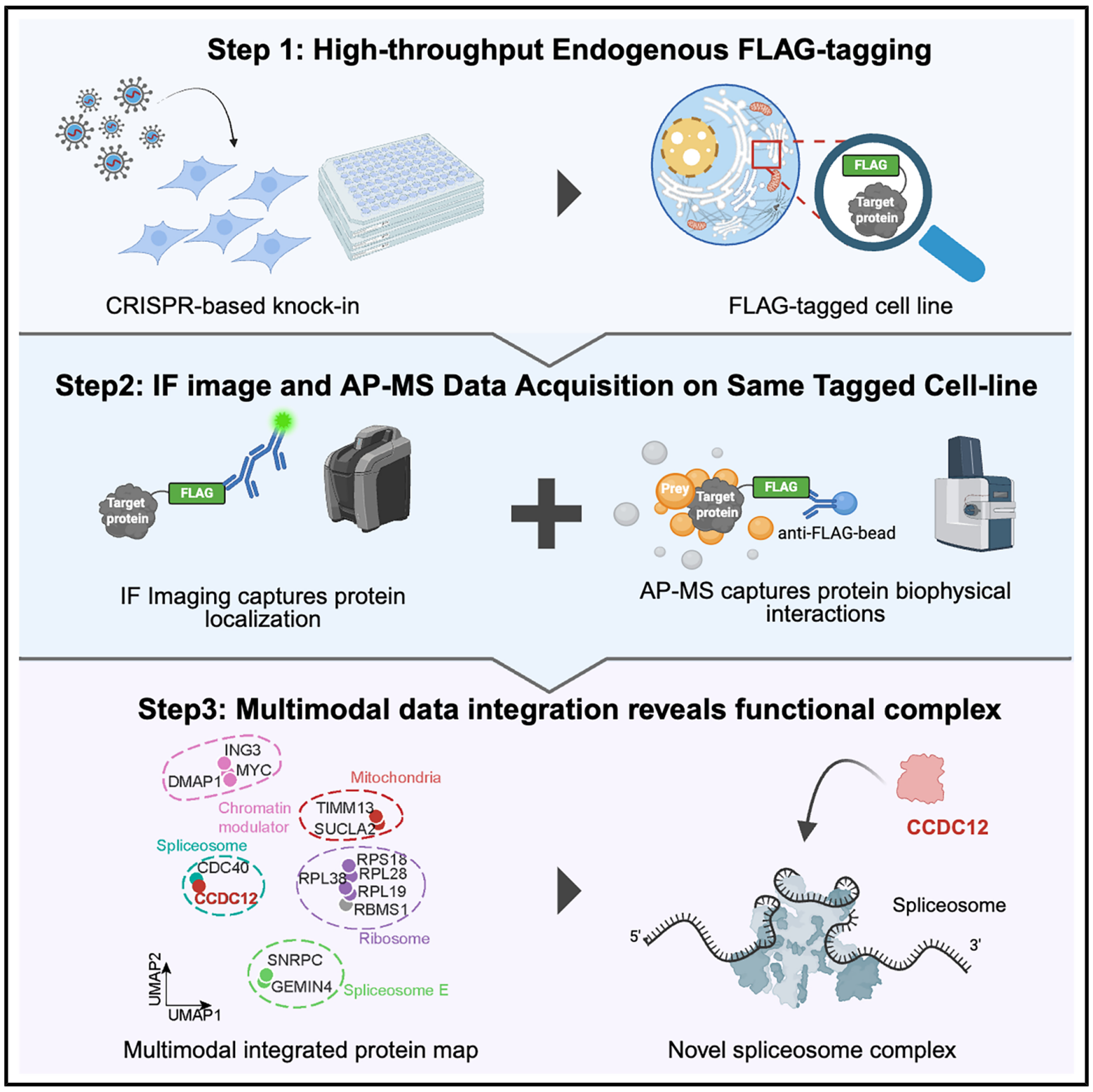

Highlights

- HIT-MAP couples endogenous protein tagging with IF imaging and AP-MS
- 9×FLAG improves detection of endogenously expressed proteins over 3×FLAG
- Deconvolved wide-field microscopy approximates confocal IF localization
- Integration nominates CCDC12 as a candidate Bact spliceosomal complex component

Authors

Gege Qian, Joonwon Kim, Richa Tiwari, ..., Nevan Krogan, Trey Ideker, Alejandro Chavez

Correspondence

tideker@health.ucsd.edu (T.I.), chavez2@health.ucsd.edu (A.C.)

In brief

Understanding how proteins organize inside cells requires combining imaging and interaction data, yet doing so at scale remains technically demanding. Qian et al. present HIT-MAP, a streamlined pipeline that tags proteins at native genomic sites and acquires both modalities from one cell line, broadening access to multimodal cell mapping.

Qian et al., 2026, Cell Reports Methods *6*, 101534
September 21, 2026 © 2026 The Authors. Published by Elsevier Inc.
https://doi.org/10.1016/j.crmeth.2026.101534

CellPress

Cell Reports Methods

CellPress
OPEN ACCESS

Article

HIT-MAP: A scalable approach to multimodal mapping of subcellular organization

Gege Qian,[1,2,11] Joonwon Kim,[3,4,11] Richa Tiwari,[5,6,11] Benjamin J. Polacco,[5,6] Antoine Forget,[5,6] Leah V. Schaffer,[2] Hark Kyun Kim,[3] Jiahao Gao,[2] Yuan Zhou,[5,6] Gwendolyn M. Jang,[5,6] Marcus R. Kelly,[2,7] Xiaoyu Zhao,[2,7] Helene Foussard,[5,6] Liudeng Zhang,[3] Nevan Krogan,[5,6,8] Trey Ideker,[2,9,10,*] and Alejandro Chavez[1,3,12,*]

[1]Bioinformatics and Systems Biology Program, University of California San Diego, La Jolla, CA, USA
[2]Department of Medicine, University of California San Diego, La Jolla, CA, USA
[3]Department of Pediatrics, University of California San Diego, La Jolla, CA, USA
[4]Department of Chemical and Biological Engineering, Seoul National University, Seoul, Republic of Korea
[5]Quantitative Biosciences Institute (QBI), University of California San Francisco, San Francisco, CA, USA
[6]Gladstone Institute of Data Science and Biotechnology, J. David Gladstone Institutes, San Francisco, CA, USA
[7]Moores Cancer Center, University of California San Diego, La Jolla, CA, USA
[8]Department of Bioengineering and Therapeutic Sciences, University of California San Francisco, San Francisco, CA, USA
[9]Department of Computer Science and Engineering, University of California San Diego, La Jolla, CA, USA
[10]Department of Bioengineering, University of California San Diego, La Jolla, CA, USA
[11]These authors contributed equally
[12]Lead contact
*Correspondence: tideker@health.ucsd.edu (T.I.), chavez2@health.ucsd.edu (A.C.)
https://doi.org/10.1016/j.crmeth.2026.101534

MOTIVATION Proteomic technologies, particularly immunofluorescence (IF) imaging and affinity purification-mass spectrometry (AP-MS), have transformed our ability to map subcellular organization, but generating multimodal datasets at scale remains restricted to consortia with specialized infrastructure. Conventional workflows depend on target-specific antibodies or cDNA overexpression, which scale poorly in cost and labor, can fail to capture native protein expression and splicing, and often require separate detection strategies for obtaining imaging and interaction data. Existing endogenous tagging pipelines address some of these issues but rely on gene-specific donor constructs and compartment-restricted fluorescent tags, while leaving the systematic optimization of the underlying data collection pipeline largely unexplored. Here, we developed high-throughput integrated tagging for cell mapping (HIT-MAP), an end-to-end pipeline that couples high-throughput endogenous epitope tagging with readily accessible wide-field imaging and an optimized approach to capturing native protein complexes, enabling coordinated multimodal data acquisition with substantially reduced cost and operational complexity.

SUMMARY

Multimodal mapping of subcellular protein organization through imaging and interaction proteomics has been mostly confined to large consortia, owing to high per-target costs, reliance on target-specific antibodies or libraries of epitope-tagged cDNAs, and the lack of unified pipelines for coordinated data acquisition. Here, we present HIT-MAP (high-throughput integrated tagging for cell mapping), an end-to-end framework that couples endogenous epitope tagging with optimized wide-field immunofluorescence imaging and affinity purification-mass spectrometry (AP-MS). Applied to a pilot set of 16 representative proteins in HEK293T cells, HIT-MAP recovers 576 high-confidence protein-protein interactions, identifies canonical complexes, and resolves cross-modality protein communities. Integrative analysis identifies CCDC12 as a previously uncharacterized component of the Bact spliceosomal complex, supported by AP-MS interaction data, tag-free size-exclusion chromatography-mass spectrometry (SEC-MS) co-fractionation, and Perturb-seq transcriptional signatures. HIT-MAP lowers technical and economic barriers to generating coordinated multimodal protein maps.

© 2026 The Authors. Published by Elsevier Inc.
This is an open access article under the CC BY license (http://creativecommons.org/licenses/by/4.0/).

INTRODUCTION

Recent technological advances allow for the collection of proteomic data over a broad range of scales and resolutions. Among the various data types, subcellular protein imaging and biophysical interaction have been repeatedly shown to be highly informative of proteome organization and function. Large efforts have therefore generated extensive datasets using immunofluorescence (IF) and affinity purification-mass spectrometry (AP-MS) across diverse cell types.[1–4] Recently, we and others have demonstrated that integration of imaging and interaction data enables construction of multiscale cell maps that reveal subcellular compartments and protein complexes *de novo* that can serve as a reference for understanding subcellular organization.

Despite these advances, generating such multimodal maps remains technically and economically demanding. Large-scale cell mapping has largely been restricted to major consortia with dedicated infrastructure, limiting broader adoption. Confocal microscopy, the prevailing standard for IF imaging, provides high-resolution optical sectioning but requires costly instrumentation, specialized expertise, and, in many cases, may not have the required add-ons to enable automated image acquisition. Similarly, large-scale AP-MS experiments face the challenge of distinguishing bona fide interactors from abundant contaminants or non-specific binders. Generating cDNA constructs to express the target protein at supraphysiological levels is one way to improve detection sensitivity. However, this not only risks capturing spurious interactions but also fails to provide information on protein isoforms that may be critical for native interaction dynamics and building accurate cell-type-specific maps.[5,6]

A further bottleneck is that large-scale imaging and AP-MS datasets are typically generated independently, using different protein detection strategies, leading to duplication of effort and increased per-target cost. Endogenous tagging offers a promising solution by enabling standardized protein detection across analytical platforms (e.g., IF and AP-MS) from a single clonal cell line. Several large-scale endogenous tagging efforts have demonstrated the feasibility of this approach.[7–9] OpenCell,[10] for example, generated high-quality imaging and interaction datasets for many human proteins using fluorescent tags and arrayed CRISPR knockin strategies. However, arrayed approaches require gene-specific donor constructs, individual transfections, and extensive cell sorting for each target, creating substantial bottlenecks for implementation outside specialized settings. Moreover, fluorescent tags such as split-GFP require compartment-specific expression of complementary fragments, limiting their utility for proteins residing in inaccessible organelles. Critically, while these efforts have demonstrated what is possible at scale, the optimization of the underlying data collection pipeline—including tag selection, imaging platform, and interaction scoring—has received comparatively little systematic attention, particularly in the context of proteins expressed at physiological levels where signal sensitivity is most limiting. To address these limitations, we recently developed HITAG, a pooled CRISPR-non-homologous end joining (NHEJ)-based method for high-throughput endogenous tagging that uses a universal donor construct and pooled cell manipulation to rapidly generate libraries of tagged clones.[11] While HITAG enables scalable tagging, a streamlined end-to-end framework for converting such tagged cell lines into integrated spatial and interaction maps requires detailed benchmarking and optimization across the captured modalities to ensure its validity.

Here, we introduce high-throughput integrated tagging for cell mapping (HIT-MAP), an integrated pipeline that systematically optimizes this process. Specifically, (1) we benchmark epitope tag valency to improve detection sensitivity at endogenous expression levels, (2) demonstrate that computationally deconvolved wide-field microscopy can accurately capture protein localization data while enabling higher throughput and more accessible imaging, and (3) provide a standardized framework for integrating imaging and interaction data into multimodal protein embeddings. As a proof of concept, we apply HIT-MAP to 16 representative proteins in HEK293T cells, demonstrating the complete workflow from clone generation through multimodal data acquisition and integrated co-embedding. This pilot establishes the feasibility of a scalable, unified data generation pipeline that lowers barriers to large-scale spatial proteomics and, despite its limited scale, reveals CCDC12 as a previously uncharacterized component of the human spliceosomal complex—illustrating the capacity of HIT-MAP to refine functional annotation even in small datasets.

RESULTS

Overview of the HIT-MAP pipeline and consideration of an appropriate epitope tag

To establish the HIT-MAP workflow, we leveraged our previously published endogenous tagging strategy, HITAG,[11] which enables rapid insertion of epitope tags at native genomic loci using CRISPR-Cas9-mediated non-homologous end joining. As an initial step, we generated a panel of endogenously tagged HEK293T cell lines that served as standardized inputs for both imaging and interaction profiling (Figure 1). Because HIT-MAP is designed to support multimodal data acquisition, we sought an epitope tag configuration compatible with both IF imaging and AP-MS. Although 3×FLAG is widely used in overexpression-based systems, its sensitivity for detecting proteins expressed at physiological levels has not been extensively investigated. We therefore directly compared two tag valencies—3×FLAG and 9×FLAG, comprising three or nine tandem FLAG epitopes—to evaluate whether increased epitope density improves signal-to-noise in both IF and AP-MS without perturbing protein behavior.

As a proof-of-concept pilot, we targeted 16 proteins spanning three major subcellular compartments (nucleus, cytosol, and mitochondria). For each protein, independent cell lines harboring either 3×FLAG or 9×FLAG C-terminal insertions were generated using an arrayed version of our HITAG strategy. Successful tag integration was validated by PCR across the insertion site and Sanger sequencing (Figures S1 and S2). Western blot analysis using antibodies against FLAG confirmed expression of the tagged proteins at the expected molecular weight (Figures S3–S5). No evidence of gross overexpression relative to parental HEK293T cells was observed. Together, these validated cell lines enabled systematic benchmarking of tag performance

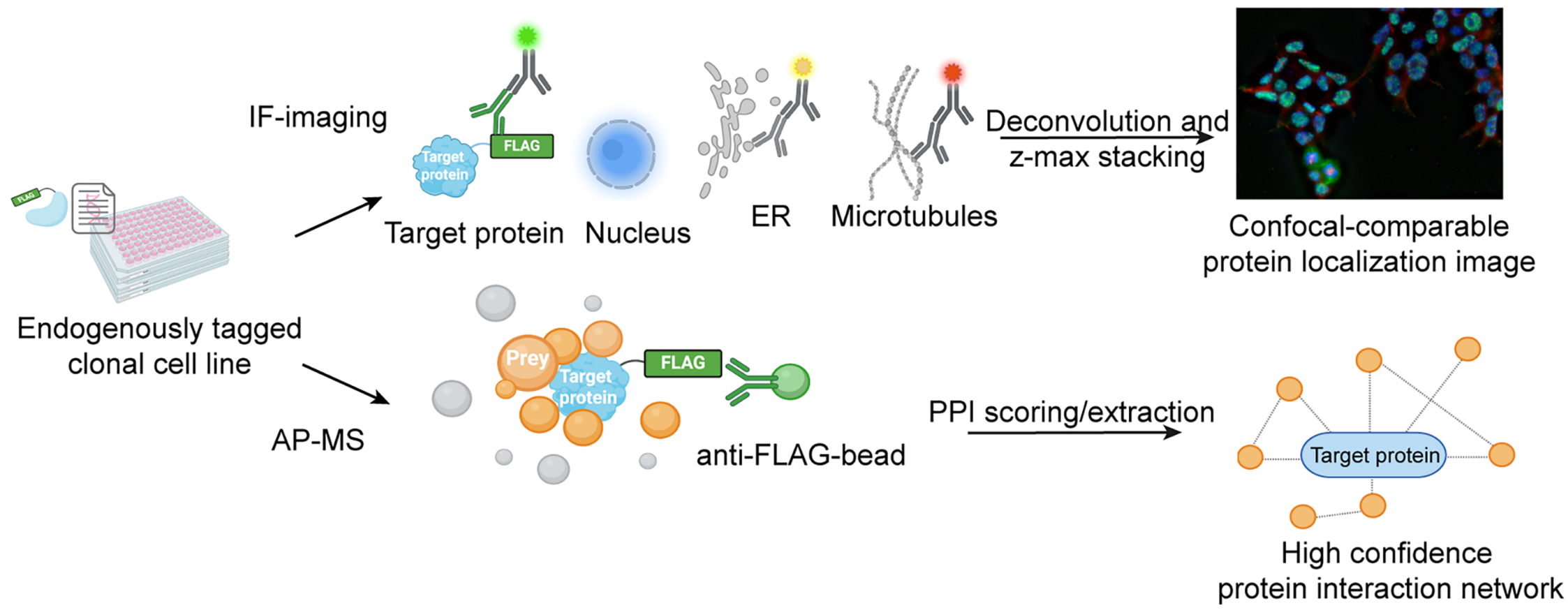

Figure 1. Accelerated pipeline for multimodal proteomic data collection using HIT-MAP

Arrays of FLAG-tagged clonal cell lines are constructed using HITAG. The cell lines are suitable for both immunofluorescence imaging and AP-MS experiments using anti-FLAG antibodies. For the immunofluorescence imaging, cells are stained for nucleus (DAPI), microtubules (anti-β-tubulin, Alexa Fluor 647), ER (anti-CALR, Alexa Fluor 555), and the protein of interest (anti-FLAG, Alexa Fluor 488) and then imaged using wide-field microscopy at different depths. Obtained images are projected through the z axis and further computationally deconvolved to remove out-of-focus light noise to generate confocal-comparable images. In parallel, the same clonal cell line is used for FLAG tag-based affinity purification followed by mass spectrometry; protein-protein interactions are then scored and filtered by confidence.

across imaging and interaction modalities under endogenous expression conditions.

Deconvolved wide-field microscopy approximates confocal localization measurements

To evaluate whether automated wide-field microscopy could support scalable IF acquisition within HIT-MAP, we systematically compared wide-field and confocal imaging across all tagged proteins (Figure 2A). For each cell line, 31 optical sections were acquired using a Keyence BZ-X800 microscope and collapsed via maximum intensity projection to preserve three-dimensional cellular structure (STAR Methods). Four fluorescence channels were recorded per sample: nuclear, microtubule, and endoplasmic reticulum (ER) landmarks, along with the FLAG-tagged protein of interest. Unlike confocal microscopy, wide-field imaging captures substantial out-of-focus light, potentially obscuring subcellular localization patterns. To mitigate this effect, we applied computational deconvolution to each z stack using the Richardson-Lucy iterative algorithm and canonical point spread functions (PSFs) (STAR Methods).[12] While empirical PSF measurements can further improve optical modeling, canonical PSFs provide a practical and widely used approximation for high-throughput imaging workflows. The resulting images were processed using a DenseNet-121 convolutional neural network previously trained on confocal IF datasets,[13] generating 1,024-dimensional embedding vectors that capture protein localization signatures (Figure 2A; STAR Methods).

We first confirmed that endogenous tagging largely preserved expected localization patterns using in-house confocal imaging of both 3×FLAG- and 9×FLAG-tagged lines. For the majority of proteins, localization patterns were concordant with prior knowledge. However, several targets—including CCDC12, ING3, TOMM40, and TIMM13—exhibited weaker and more diffuse signal when tagged with 3×FLAG, complicating localization assignment (Figures S6A and S6B). These observations motivated a systematic comparison of tag valency and imaging modality. To quantitatively benchmark imaging performance, we compared embedding similarity between in-house images (confocal, raw wide-field, and deconvolved wide-field) and reference confocal images from the Human Protein Atlas (HPA)[14] (Tables S1–S6). HPA images, generated using antibodies against endogenous proteins, served as an independent reference for localization.

Across the 16 proteins examined, 9×FLAG-tagged samples exhibited higher median embedding similarity to HPA references than 3×FLAG-tagged samples, although this difference did not reach conventional statistical significance in this pilot dataset (paired Student's *t* test, $p = 0.092$; Figure 2B). A consistent trend of improvement or similar quality can be observed for most proteins in our experiment. These results suggest that increased epitope valency can improve signal-to-noise under endogenous expression levels.

Importantly, deconvolution substantially improved wide-field image fidelity. While raw wide-field embeddings showed reduced similarity to HPA references relative to confocal imaging, deconvolved wide-field images demonstrated significantly increased concordance (paired Student's *t* test, $p = 0.0007$; Figures 2C and 2D; Figure S6C). In this pilot set, deconvolved wide-field embeddings approached the similarity levels observed for confocal imaging, indicating that computational correction can partially compensate for the optical limitations of wide-field microscopy. We note that this comparison was performed across a limited and diverse set of proteins, and further evaluation on proteins with highly punctate or fine subcellular structures will be required to comprehensively assess performance boundaries. Nevertheless, these results support the feasibility of wide-field microscopy, when coupled with deconvolution, as a scalable alternative for large-scale localization profiling within the HIT-MAP workflow.

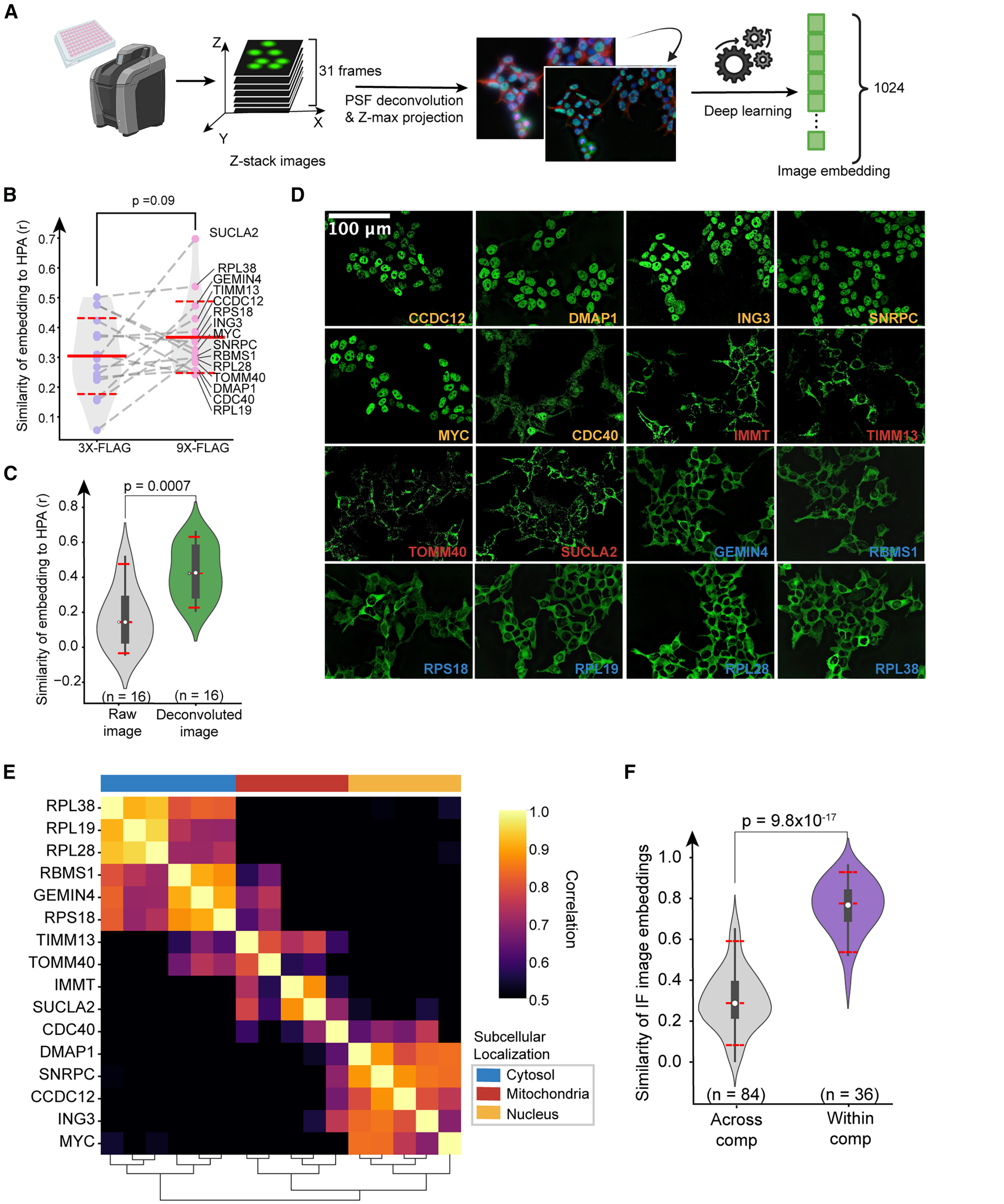

Figure 2. Pilot IF imaging and comparison to current standards for localization characterization

(A) IF imaging pipeline.

(B) Comparison of the distribution of correlation values between each protein's confocal immunofluorescence (IF) image and the corresponding Human Protein Atlas (HPA) reference image under the 3×FLAG- or 9×FLAG-tagging condition (p = 0.09 by paired Student's t test).

(C) Comparison of the correlation between wide-field microscopy image embeddings and HPA reference image embeddings for each protein (p = 0.0007 by paired Student's t test). Correlations are shown for raw *Z*max projected images (gray) and deconvolved images (green) of 9×FLAG-tagged proteins.

(legend continued on next page)

CellPress
OPEN ACCESS

To assess whether image embeddings captured biologically meaningful spatial information beyond technical benchmarking, we examined pairwise similarities among proteins. Embeddings clustered proteins according to their annotated subcellular compartments, with clear separation of nuclear, cytosolic, and mitochondrial groups (Figure 2E). Proteins within the same compartment exhibited significantly higher embedding similarity compared to proteins from different compartments (Mann-Whitney U test, $p = 9.8 \times 10^{-17}$; Figure 2F), demonstrating that the learned representations encode biologically relevant spatial features.

Endogenous AP-MS recovers high-confidence biophysical interaction networks

Using the same 32 endogenously tagged cell lines generated for imaging (16 proteins $\times$ 2 tag valencies), we performed AP-MS profiling with four biological replicates per bait using a data-dependent acquisition (DDA) workflow (STAR Methods). High-confidence protein-protein interactions (PPIs) were identified through statistical scoring and contaminant filtering prior to network construction. To capture higher order network structure, bait interaction profiles were embedded into a 1,024-dimensional feature space using Node2Vec[15] (Figure 3A). To systematically evaluate the impact of epitope tag valency on interaction recovery, we benchmarked AP-MS data from 3$\times$FLAG- and 9$\times$FLAG-tagged baits against three independent, curated protein interaction databases: CORUM,[16] STRING,[17] and hu.MAP 2.0.[18] Area under the receiver operating characteristic (AUROC) analysis revealed that 9$\times$FLAG-tagged baits consistently and substantially outperformed their 3$\times$FLAG counterparts across all three reference datasets, achieving AUROC values of 0.797, 0.874, and 0.968 versus 0.707, 0.686, and 0.754, respectively (Figure 3B). This convergent evidence across orthogonal reference frameworks indicates that the increased epitope density conferred by 9$\times$FLAG enhances antibody-mediated capture efficiency at endogenous expression levels, where epitope accessibility and stoichiometry are likely limiting. Together, these data establish 9$\times$FLAG as the preferred tag configuration within the HIT-MAP pipeline, particularly for proteins expressed at low-to-moderate abundance where maximizing pull-down sensitivity is critical for faithful recovery of native interaction networks.

After applying stringent confidence thresholds (STAR Methods), we identified 576 PPIs among 331 proteins (Figures 3C and 3E; Figure S7A, Tables S7–S9). Of these, 62.5% (360/576) overlapped with STRING or CORUM annotations—meeting or exceeding the 3%–25% cross-study reproducibility typical of large-scale AP-MS datasets.[10] To assess whether the remaining 37.5% of interactions represent genuine undocumented associations rather than experimental artifacts, we sought orthogonal evidence from two independent proteome-wide resources: a pan-cancer co-abundance network derived from quantitative proteomic profiling of 949 human cell lines and a proteome-wide co-regulation map capturing coordinated protein-level responses across hundreds of biological conditions. For each resource, we constructed a high-confidence protein interaction graph and computed the shortest path length between AP-MS-detected protein pairs within that graph. Novel HIT-MAP interactions were significantly more proximal than randomly selected protein pairs in both frameworks (Mann-Whitney U test, $p = 5.6 \times 10^{-20}$ and $p = 2.4 \times 10^{-17}$, respectively; Figures S7B and S7C), with path length distributions shifted toward those of known curated interactions. The convergent support across two biologically independent, proteome-scale references argues strongly that these novel PPIs reflect genuine physical associations not yet captured in curated databases, rather than non-specific contaminants, and highlights the unique capacity of endogenous-expression AP-MS to expand the known human interactome.

Consistent with biological expectation, canonical complexes—including the ribosome, spliceosome, SMN complex, and NuA4-TIP60 histone acetyltransferase complex—were robustly recapitulated, with bait proteins serving as central hubs within coherent subnetworks (Figure 3E). Ribosomal proteins such as RPL28 and RPL19 exhibited the highest number of interactors, consistent with their roles as scaffold components of large multiprotein assemblies. In contrast, mitochondrial baits yielded fewer high-confidence interactions: TOMM40 failed to recover detectable prey and was excluded from downstream analysis (STAR Methods; Figure S7D), while IMMT and SUCLA2 produced limited prey at stringent thresholds, though relaxed cutoffs enriched for known mitochondrial interactors (Figure S7E). This reduced recovery likely reflects the well-documented challenges of endogenous AP-MS for membrane-embedded or low-abundance proteins, rather than a failure of tagging, as analogous limitations have been reported in other large-scale endogenous-tag interaction maps (Figure S7F).

To further assess biological plausibility, we examined subcellular localization concordance between bait and prey proteins. Interactions were significantly enriched for same-compartment associations (Figures S7G and S7H), supporting physiological relevance of the recovered network. Moreover, recovery of curated CORUM complexes was comparable to prior AP-MS resources such as BioPlex and OpenCell (Figure 3D), indicating that endogenous tagging combined with standardized affinity purification can recover interaction networks of similar quality. Together, these results demonstrate that HIT-MAP enables recovery of biologically coherent PPI networks from endogenously tagged baits, while preserving native expression context.

Identification of protein communities with cross-modality support

Building on our previously developed MuSIC framework,[19,20] we integrated IF- and AP-MS-derived embeddings for the 14

(D) Representative deconvolved 9$\times$FLAG-tagged IF images of the 16 tagged proteins, with protein names labeled at the bottom right of each image. Protein label color corresponds to their known subcellular localization: nuclear (yellow), mitochondrial (red), and cytosolic proteins (blue).
(E) Clustering of deconvolved 9$\times$FLAG-tagged protein's IF image embeddings for all 16 proteins. The top color bar indicates compartment annotation from HPA.
(F) Pairwise cosine similarity of deconvolved wide-field image embeddings among proteins within the same subcellular compartment and across different compartments ($p = 9.8 \times 10^{-17}$ by Mann-Whitney U test).

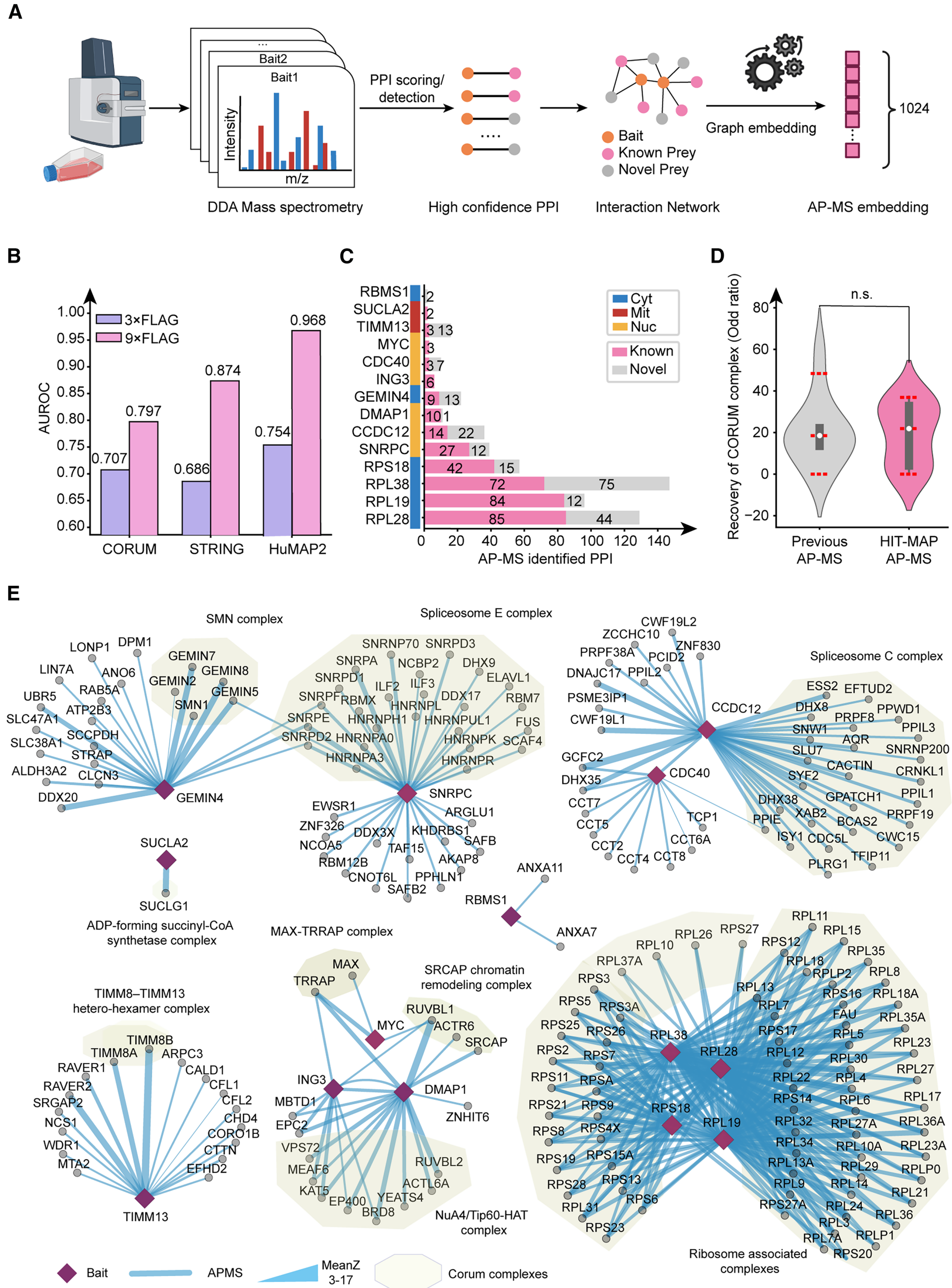

Figure 3. HIT-MAP robustly captures native PPI

(A) AP-MS data generation and processing pipeline.

(B) Receiver operating characteristic (ROC) curves comparing the enrichment of protein-protein interactions (PPIs) identified using 3×FLAG and 9×FLAG tags against reference PPI databases STRING (combination score >600) and CORUM.

(C) Identified PPIs by baits. Preys are annotated as known (pink) (referencing STRING and CORUM database) and novel (gray).

(legend continued on next page)

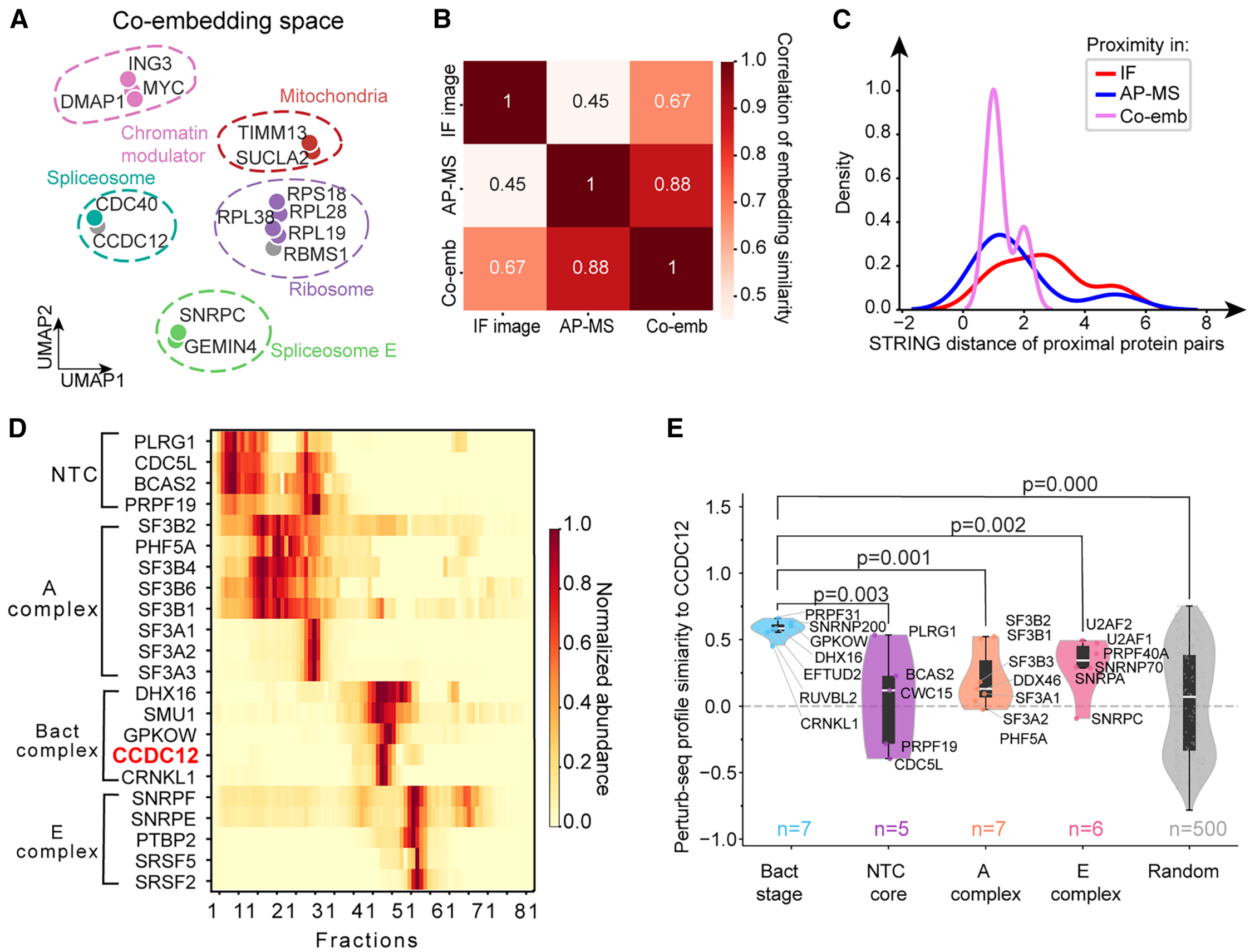

Figure 4. Multimodal embedding and functional annotation of pilot bait proteins

(A) UMAP plots showing the integrated multimodal embedding of IF and AP-MS modalities reveal a joint representation that incorporates both imaging and interaction information. Clusters are labeled based on the members within a known complex. Of the 16 analyzed proteins, 14 had IF and high-confidence AP-MS interactions and are plotted.

(B) Heatmap showing the Pearson correlation between pairwise protein similarity matrices derived from the three embedding spaces: IF image-based embeddings, AP-MS PPI network-based embeddings, and integrated multimodal embeddings. Each matrix captures the pairwise similarity between the 16 pilot bait proteins within a given modality.

(C) Distribution of STRING PPI-network distances for protein pairs with high embedding similarity (cosine similarity >0.6) across three data modalities: IF image-based embeddings, AP-MS PPI network-based embeddings, and integrated multimodal embeddings.

(D) SEC-MS elution profiles of CCDC12 and representative spliceosome complex proteins across 81 size-exclusion chromatography fractions in unmodified HEK293 cells.[22] Proteins are grouped by their established spliceosomal complex membership (Bact, NTC core, A complex, and E complex). Normalized abundance values are shown as a heatmap, with peak co-elution of CCDC12 with Bact complex components in high-molecular-weight fractions providing tag-free, endogenous evidence for CCDC12's association with the activated spliceosome.

(E) Perturb-seq transcriptional profile[23] similarity between CCDC12 and members of distinct spliceosomal complexes (Bact, NTC core, A complex, and E complex) relative to 500 randomly selected genes, derived from genome-wide Perturb-seq data in HEK293T cells.

Each data point represents the similarity score between the transcriptional response to CCDC12 knockdown and that of individual spliceosomal complex members (Mann-Whitney U test).

proteins represented in both modalities (Tables S10–S12). Unlike prior work that relied on pre-existing datasets, this integration was performed on data generated within a single standardized tagging and acquisition pipeline, allowing direct assessment of multimodal coherence within HIT-MAP. Embeddings derived independently from IF images, AP-MS interaction profiles, and their multimodal integration were visualized using uniform manifold approximation and projection (UMAP)[21] (Figure 4A; Figures S8A and S8B). As expected, IF embeddings primarily grouped proteins according to subcellular localization,

(D) Distribution of odds ratios for enrichment of AP-MS-detected PPIs within CORUM protein complexes. Odds ratios are computed based on the overlap between experimentally detected PPIs of baits in HEK293T cells from other studies (BioPlex 3.0 and OpenCell) and this study and curated interactions in CORUM ($p = 0.9209$, Student's t test).

(E) AP-MS-detected PPI network, highlighting CORUM complexes recovered for each bait. For PPI mapping, a cutoff of mean $Z_{MADEX} \geq 3$ and $p \leq 0.01$ is applied. For the ribosomal proteins, a partial network highlighting recovered ribosomal subunits is represented here; the rest is shown in Figure S7B. Preys within known CORUM complexes of each bait are annotated with corresponding colors.

whereas AP-MS embeddings grouped proteins by shared physical interactions. The integrated multimodal embedding combined these complementary signals, resolving compartment-restricted substructures in cases where interaction evidence alone was limited. For example, mitochondrial proteins with sparse AP-MS interactions clustered together in the integrated space due to strong spatial concordance.

To quantify the degree to which modality-specific information was retained following integration, we computed Pearson correlations between pairwise protein distances in the multimodal embedding and those derived from IF and AP-MS individually. While IF and AP-MS embeddings were only moderately correlated with one another, the multimodal embedding showed substantial similarity to both (Figure 4B), indicating that integration preserved key spatial and interaction features. Moreover, protein pairs that were proximal in the multimodal space were significantly more likely to share functional associations in the STRING network compared to pairs close in either modality alone (Figure 4C). These findings suggest that multimodal integration enhances recovery of known functional relationships, even within a limited pilot set. Within the integrated embedding, proteins formed five discrete clusters corresponding to known biological assemblies: ribosomal proteins (RPS18, RPL28, RPL19, RPL38, and RBMS1), spliceosome E complex components (SNRPC and GEMIN4), spliceosome complex proteins (CDC40 and CCDC12), chromatin modulators (ING3, MYC, and DMAP1), and mitochondrial proteins (TIMM13 and SUCLA2) (Figure 4A). While many of these groupings reflect established biology, the multimodal embedding clarified relationships in cases where one modality alone was insufficient. For instance, TIMM13 and SUCLA2 clustered together primarily through shared spatial localization despite limited AP-MS recovery. Conversely, RBMS1 co-localized with ribosomal proteins without sharing detected physical interactions, suggesting spatial proximity without stable complex membership.

The integrated embedding also linked CCDC12, a poorly characterized protein in human cells, with splicing. Although CCDC12 has been implicated in spliceosomal biology in *C. elegans*,[24,25] its role in human systems remains undefined. In our multimodal embedding, CCDC12 clustered closely with CDC40, a core spliceosome C complex component (Figure 4A), prompting systematic investigation through three orthogonal lines of evidence. In our AP-MS data, CCDC12 pulled down an extensive network of high-confidence interactors with striking specificity for spliceosomal machinery, predominantly comprising established Bact and C complex components—including ESS2, EFTUD2, DHX8, PRPF8, AQR, BCAS2, CWC15, CRNKL1, and PLRG1—many of which orchestrate the Bact-to-C complex catalytic transition (Figure 3E). To validate this association on the untagged endogenous protein, we interrogated proteome-wide size-exclusion chromatography-mass spectrometry (SEC-MS) data from unmodified HEK293 cells,[22] finding that CCDC12 co-elutes with Bact complex components—SMU1, DHX16, GPKOW, and CRNKL1—at molecular weights consistent with the spectrum of spliceosomal complexes (Figure 4D). Finally, genome-wide Perturb-seq[23] analysis revealed that the transcriptional signature of CCDC12 knockdown was significantly more similar to perturbations of Bact complex members than to those of the NTC core, A complex, E complex, or 500 random genes (Mann-Whitney U test, p = 0.003, 0.001, 0.002, and $p < 0.0001$, respectively; Figure 4E). The convergence of AP-MS interaction specificity, endogenous co-fractionation, and transcriptional perturbation signatures across three independent datasets collectively argues that CCDC12 is a previously uncharacterized component of the human Bact spliceosomal complex, likely contributing to the Bact-to-C complex transition during pre-mRNA splicing catalysis. We note that this pilot integration encompasses a limited and intentionally diverse set of proteins. While not sufficient to establish large-scale organizational principles, these results demonstrate that data generated within the HIT-MAP pipeline can be coherently integrated across modalities and can refine functional annotation even in small datasets.

DISCUSSION

In this study, we present HIT-MAP, an integrated workflow that streamlines endogenous tagging, imaging, and interaction proteomics into a unified and standardized pipeline for multimodal protein characterization. Rather than introducing a new data modality, HIT-MAP focuses on optimizing the upstream data generation process to improve reproducibility, reduce per-target cost, and facilitate coordinated acquisition of imaging and interaction data from the same tagged cell lines.

Several features distinguish HIT-MAP from conventional protein characterization workflows (Figures 5A and 5B). First, by leveraging the HITAG endogenous tagging strategy, which utilizes a universal donor construct and is compatible with pooled CRISPR-based integration, the time and labor required to generate tagged cell lines are reduced relative to arrayed, gene-specific donor approaches. Importantly, tagging at the endogenous locus preserves native expression and splicing, enabling interrogation of protein behavior under physiological conditions without reliance on overexpression systems. Second, the use of a standardized epitope tag across targets allows the same detection reagents and experimental protocols to be applied across both IF and AP-MS. This unified detection strategy minimizes variability introduced by protein-specific antibodies and facilitates direct multimodal integration from a single clonal cell line. Third, we demonstrate that automated wide-field microscopy, when coupled with computational deconvolution and deep-learning-based embedding, can approximate confocal-derived localization measurements in this pilot dataset. While confocal microscopy remains advantageous for resolving fine subcellular structures, wide-field imaging offers practical advantages in automation and throughput that may be particularly relevant for large-scale studies. Together, these elements form a cohesive pipeline that standardizes multimodal data acquisition at endogenous expression levels. Finally, we note that, when tagging hundreds of targets, using a pooled tagging strategy is most efficient, as this greatly streamlines the time, labor, and reagent costs. However, when examining smaller numbers of proteins, an array-based approach to tagging, such as the one taken in this manuscript, can also be used.

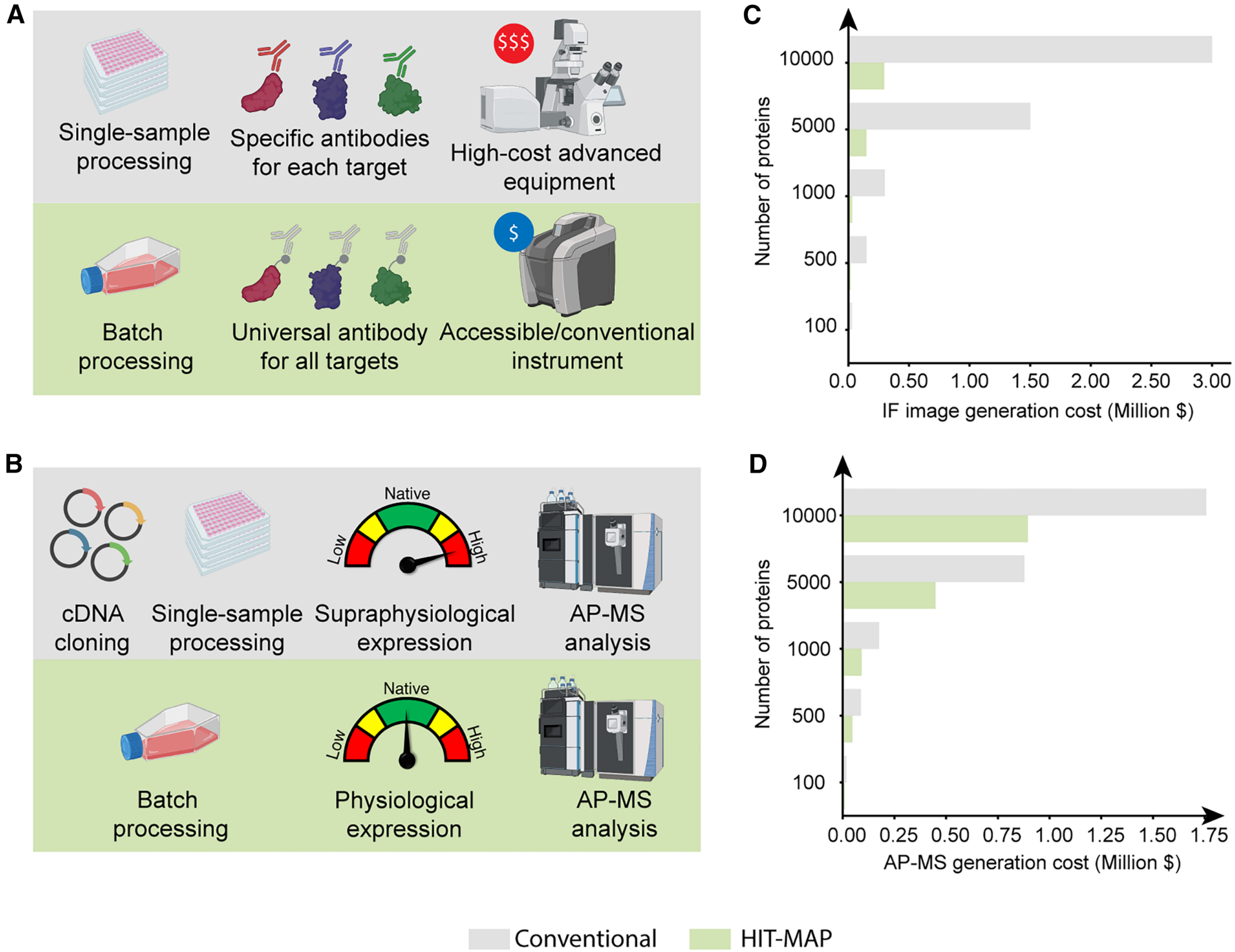

Figure 5. Comparison between the conventional method and the streamlined HIT-MAP pipeline
(A and B) Comparison of resource requirements between conventional approaches and the HIT-MAP pipeline for (A) image generation and (B) AP-MS analysis.
(C and D) Cost analysis graphs of the HIT-MAP pipeline (green) compared to conventional methods (gray) across increasing numbers of protein targets.

Compared to prior endogenous tagging resources such as OpenCell, the outlined studies emphasize workflow modularity and experimental standardization rather than the generation of a comprehensive atlas. While arrayed tagging platforms have successfully produced large-scale datasets, they typically require gene-specific donor constructs, individualized cloning steps, and specialized automation infrastructure. In contrast, the strategy underlying HIT-MAP consolidates tagging into a shared workflow that can be deployed with more modest laboratory resources (Figures 5C and 5D; Figure S9). As such, HIT-MAP is intended to complement, rather than replace, existing large-scale efforts by lowering operational barriers for laboratories seeking to generate coordinated multimodal datasets in defined biological contexts.

The economic superiority of the system arises from no longer requiring target-specific antibodies, for which costs and effort scale linearly with the number of targets, by using a universal epitope tag and a single well-validated antibody against the epitope that provides increasing cost efficiency as the target library expands compared to conventional methodologies (Tables S13–S16). Even with conservative estimates that include the total cost of clonal cell line generation, characterizing 10,000 proteins via HIT-MAP is approximately 10-fold less expensive than requiring a unique antibody for each target being explored and roughly half the cost of cDNA-overexpression systems. In addition, integration of wide-field imaging increases data acquisition throughput per instrument-hour, effectively decoupling the temporal and labor burden from expanding study scale. This efficiency is further improved in multimodal studies, as the initial cost of generating a single clonal cell line is shared across both imaging and interaction mapping.

Beyond these financial advantages, HIT-MAP provides distinct operational and technical benefits over arrayed tagging platforms such as OpenCell (Tables S16–S17). While arrayed methods rely on target-specific donor templates, nucleotide synthesis for individual targets, and automation platforms to manage the generation of individualized clones, our pooled strategy employs a universal donor system to consolidate labor-intensive requirements into a single-vessel workflow with a considerably less expensive system. Our operational analysis indicates that this parallelized architecture increases throughput per man-hour, as it avoids the linear accumulation of labor typically associated with large-scale genetic engineering. Additionally, our tagging strategy offers greater versatility across diverse

organelles as it does not depend on the compartment-specific expression requirements often associated with split-protein systems such as those used by OpenCell. By lowering financial and operational barriers, HIT-MAP enables comprehensive subcellular mapping in a broader range of laboratory settings.

Despite the significant advantages presented, the use of endogenous gene tagging has inherent limitations that necessitate careful consideration during the analysis of the resulting data. Previous studies have found that tagging a protein can alter its subcellular distribution, which can have a direct impact on the proteins with which it can interact.[10,26] When studies have compared data obtained with epitope-tagged human proteins versus antibodies against the native human proteins, approximately 80% of tagged proteins were found to overlap in their subcellular localizations.[11] These results suggest that the addition of even short peptide tags may perturb protein behavior, although it is also possible that some of the antibodies against the native human protein suffered from a lack of target specificity, a problem well documented in the literature.[27] To address this constraint, we propose using the wealth of existing biological data as an essential reference to support experimental observation. In addition, while we have used C-terminal tagging, we note that other high-throughput methods of endogenous tagging exist, such as those that insert a synthetic exon into the intron of a gene in order to splice the tag into the protein within its coding sequence.[28,29] Should C-terminal tagging be found to be detrimental for a given protein, insertion of the tag into other regions of the protein using intron-based tagging methods should be considered. Along with the potential for tagging to affect protein function, not all cell lines are equally amenable to high-throughput genetic engineering. Methods such as HITAG require highly efficient uptake of exogenous donor DNA and the required CRISPR machinery, which to date has been mainly feasible in well-established immortalized cell lines (e.g., HCT116, HEK293T, and HAP1). However, the recent explosion of diverse methods of nucleic acid and ribonuclear particle delivery suggests that these limitations may soon be solved, opening the possibility of applying approaches similar to the one outlined here to both primary cells and *in vivo* contexts.

Our proof-of-principle analysis of 16 proteins demonstrates that data generated within the HIT-MAP framework can be coherently integrated across modalities, recapitulate canonical complexes, and refine functional annotation. While the scale of this pilot dataset does not permit comprehensive mapping of cellular organization, it establishes the feasibility of an end-to-end standardized pipeline for multimodal proteomic mapping and shows the potential to guide the characterization of poorly studied proteins such as CCDC12. Furthermore, tag optimization combined with computational deconvolution enabled reliable localization of lowly expressed proteins (e.g., SUCLA2 and TOMM40), proving the efficacy of the framework even for challenging imaging targets. As the number of profiled proteins increases, we anticipate that interaction networks will be more densely connected, spatial embeddings will resolve finer subcellular distinctions typically difficult to achieve with wide-field imaging, and integrated models will support hierarchical analyses of protein organization.

By focusing on workflow optimization and multimodal standardization, HIT-MAP provides a practical framework for generating coordinated imaging and interaction datasets at endogenous expression levels. We envision that this approach will enable broader participation in spatial proteomics efforts and facilitate targeted mapping of biological systems beyond large consortium-scale initiatives.

Limitations of the study

While our goal was to develop an optimized pipeline for capturing multimodal data, some limitations are worth noting. First, we did not determine the efficiency of tag integration across our cell lines (e.g., monoallelic vs. biallelic integration). Because HITAG uses a single drug marker for selection, should the epitope tag get knocked into more than one copy of an allele, this cannot be readily distinguished. Although there is no clear mechanistic basis to expect differences between the rate at which the 3×FLAG vs. 9×FLAG tags were inserted into the genome, it remains an unmeasured variable.

Second, our pilot dataset of 16 proteins, while sufficient to benchmark the pipeline and recapitulate canonical complexes, is too limited to evaluate proteome-scale organizational principles. Moreover, tagged lines were generated individually rather than through a pooled library-scale workflow, and HIT-MAP was validated only in HEK293T cells. Several quantitative comparisons (e.g., 3×FLAG versus 9×FLAG IF similarity to HPA) did not reach conventional statistical significance. Performance on proteins with punctate, fine-structured, or membrane-embedded localizations will require further evaluation. Furthermore, mitochondrial baits in our pilot yielded limited AP-MS recovery, consistent with known challenges of endogenous-expression AP-MS for such targets.[10]

Third, our identification of CCDC12 as a Bact spliceosomal complex component is supported by three orthogonal lines of evidence (AP-MS, SEC-MS, and Perturb-seq) but lacks targeted biochemical validation, including reciprocal co-immunoprecipitation using antibodies against the native protein and functional assays for a role in the Bact-to-C transition. As such, our assignment of CCDC12 should be regarded as a strong multimodal hypothesis pending additional biochemical and structural follow-up studies.

RESOURCE AVAILABILITY

Lead contact

Requests for further information and resources should be directed to and will be fulfilled by the lead contact, Alejandro Chavez (chavez2@health.ucsd.edu).

Materials availability

The plasmids used for endogenous tagging have been deposited to Addgene (#245866–245871). Plasmids and cell samples generated in this study are available from the lead contact upon reasonable request.

Data and code availability

- The mass spectrometry proteomics data have been deposited at the ProteomeXchange Consortium via the PRIDE partner repository with the dataset identifier PRIDE: PXD067895. The confocal images, wide-field images, and deconvolved images for both 3×FLAG- and 9×FLAG-tagged cell lines of the 16 pilot proteins have been deposited

at the BioImage Archive[30] via the accession number BioImage Archive: S-BIAD2261.

- All original code has been deposited at Zenodo (Zenodo: https://doi.org/10.5281/zenodo.20548576) and is publicly available as of the date of publication. The development version is maintained at GitHub (GitHub: https://github.com/idekerlab/hit_map).
- Any additional information required to reanalyze the data reported in this paper is available from the lead contact upon request.

ACKNOWLEDGMENTS

We acknowledge funding from the Bridge2AI Program (NIH Common Fund; OT2 OD032742; T.I., N.K., and A.C.), the Cancer Cell Map Initiative (NCI Center for Cancer Systems Biology; U54 CA274502; T.I., N.K., and A.C.), the National Human Genome Research Institute (NHGRI R21HG011855; A.C.), and NIH directors fund (DP2NS131566-01; A.C.).

AUTHOR CONTRIBUTIONS

G.Q., J.K., R.T., N.K., T.I., and A.C. designed the study. G.Q., J.K., R.T., B.J.P., A.F., M.R.K., X.Z., J.G., L.V.S., N.K., T.I., and A.C. developed ideas for data analysis. J.K. generated the tagged cell lines and the image data. H.K.K. assisted with cell line generation. L.Z. generated western blot images. R.T., G.M.J., and H.F. conducted the AP-MS experiment and data collection. B.J.P., Y.Z., R.T., A.F., and G.Q. developed the pipeline and conducted the AP-MS data analysis. G.Q. implemented IF image analysis, multimodal embedding methods, computational methods, and analyses. G.Q., A.C., and T.I. led the writing of the manuscript with input from all authors.

DECLARATION OF INTERESTS

T.I. is a co-founder, advisor, and holder of equity for Data4Cure and Serinus Biosciences, and he is an advisor and shareholder for Ideaya Biosciences. A.C. has a series of CRISPR-related patents managed by Harvard and Columbia University. A.C. and J.K. have a patent on the HITAG method managed by Columbia University. A.C. is an advisor and shareholder of Syntax Bio. The terms of these arrangements for T.I. and A.C. have been reviewed and approved by UC San Diego in accordance with its conflict of interest policies. The Krogan Laboratory has received research support from Vir Biotechnology, F. Hoffmann-La Roche, and Rezo Therapeutics. N.K. has a financially compensated consulting agreement with Maze Therapeutics. N.K. is the Interim CEO and is on the Board of Directors of Mreza Therapeutics, and he is a shareholder in Mreza Therapeutics, Rezo Therapeutics, Tenaya Therapeutics, Maze Therapeutics, and GEn1E Lifesciences.

DECLARATION OF GENERATIVE AI AND AI-ASSISTED TECHNOLOGIES IN THE WRITING PROCESS

During the preparation of this work, the authors used Claude (Anthropic) in order to assist with drafting and editing manuscript text, preparing and phrasing reviewer responses, and refining the cover letter. After using this tool, the authors reviewed and edited the content as needed and take full responsibility for the content of the publication.

STAR★METHODS

Detailed methods are provided in the online version of this paper and include the following:

- KEY RESOURCES TABLE
- EXPERIMENTAL MODEL AND STUDY PARTICIPANT DETAILS
  - Plasmid construction for endogenous protein tagging
  - Generation of tagged HEK293T cell lines
- METHOD DETAILS
  - Western blot analysis
  - Acquiring IF images
  - AP-MS sample preparation
  - Protein digestion and peptide clean up
  - LC-MS/MS acquisition
  - MS database search and protein quantification
  - Data processing: Wide field image deconvolution
  - Data processing: AP-MS PPI extraction
  - Data embedding
  - Calculating costs of conventional approaches vs. HIT-MAP
- QUANTIFICATION AND STATISTICAL ANALYSIS
  - Statistical tests and significance
  - Values of n and plotting conventions
  - Significance criteria, stratification, and sample size

SUPPLEMENTAL INFORMATION

Supplemental information can be found online at https://doi.org/10.1016/j.crmeth.2026.101534.

Received: September 9, 2025
Revised: April 2, 2026
Accepted: June 25, 2026
Published: July 22, 2026

REFERENCES

1. Huttlin, E.L., Bruckner, R.J., Navarrete-Perea, J., Cannon, J.R., Baltier, K., Gebreab, F., Gygi, M.P., Thornock, A., Zarraga, G., Tam, S., et al. (2021). Dual proteome-scale networks reveal cell-specific remodeling of the human interactome. Cell *184*, 3022–3040.e28. https://doi.org/10.1016/j.cell.2021.04.011.
2. Huttlin, E.L., Bruckner, R.J., Paulo, J.A., Cannon, J.R., Ting, L., Baltier, K., Colby, G., Gebreab, F., Gygi, M.P., Parzen, H., et al. (2017). Architecture of the human interactome defines protein communities and disease networks. Nature *545*, 505–509. https://doi.org/10.1038/nature22366.
3. Huttlin, E.L., Ting, L., Bruckner, R.J., Gebreab, F., Gygi, M.P., Szpyt, J., Tam, S., Zarraga, G., Colby, G., Baltier, K., et al. (2015). The BioPlex network: a systematic exploration of the human interactome. Cell *162*, 425–440. https://doi.org/10.1016/j.cell.2015.06.043.
4. Thul, P.J., Åkesson, L., Wiking, M., Mahdessian, D., Geladaki, A., Ait Blal, H., Alm, T., Asplund, A., Björk, L., Breckels, L.M., et al. (2017). A subcellular map of the human proteome. Science *356*, eaal3321. https://doi.org/10.1126/science.aal3321.
5. Greenblatt, J.F., Alberts, B.M., and Krogan, N.J. (2024). Discovery and significance of protein-protein interactions in health and disease. Cell *187*, 6501–6517. https://doi.org/10.1016/j.cell.2024.10.038.
6. Banks, C.A.S., Boanca, G., Lee, Z.T., Florens, L., and Washburn, M.P. (2015). Proteins interacting with cloning scars: a source of false positive protein-protein interactions. Sci. Rep. *5*, 8530. https://doi.org/10.1038/srep08530.
7. Choi, W., Wu, H., Yserentant, K., Huang, B., and Cheng, Y. (2023). Efficient tagging of endogenous proteins in human cell lines for structural studies by single-particle cryo-EM. Proc. Natl. Acad. Sci. USA *120*, e2302471120. https://doi.org/10.1073/pnas.2302471120.
8. Youmans, D.T., Schmidt, J.C., and Cech, T.R. (2018). Live-cell imaging reveals the dynamics of PRC2 and recruitment to chromatin by SUZ12-associated subunits. Genes Dev. *32*, 794–805. https://doi.org/10.1101/gad.311936.118.
9. Dewari, P.S., Southgate, B., Mccarten, K., Monogarov, G., O'Duibhir, E., Quinn, N., Tyrer, A., Leitner, M.-C., Plumb, C., Kalantzaki, M., et al. (2018). An efficient and scalable pipeline for epitope tagging in mammalian stem cells using Cas9 ribonucleoprotein. eLife *7*, e35069. https://doi.org/10.7554/eLife.35069.
10. Cho, N.H., Cheveralls, K.C., Brunner, A.-D., Kim, K., Michaelis, A.C., Raghavan, P., Kobayashi, H., Savy, L., Li, J.Y., Canaj, H., et al. (2022). OpenCell: endogenous tagging for the cartography of human cellular organization. Science *375*, eabi6983. https://doi.org/10.1126/science.abi6983.

11. Kim, J., Kratz, A.F., Chen, S., Sheng, J., Kim, H.K., Zhang, L., Singh, B.K., and Chavez, A. (2024). High-throughput tagging of endogenous loci for rapid characterization of protein function. Sci. Adv. *10*, eadg8771. https://doi.org/10.1126/sciadv.adg8771.

12. Wernersson, E., Gelali, E., Girelli, G., Wang, S., Castillo, D., Mattsson Langseth, C., Verron, Q., Nguyen, H.Q., Chattoraj, S., Martinez Casals, A., et al. (2024). Deconwolf enables high-performance deconvolution of widefield fluorescence microscopy images. Nat. Methods *21*, 1245–1256. https://doi.org/10.1038/s41592-024-02294-7.

13. Ouyang, W., Winsnes, C.F., Hjelmare, M., Cesnik, A.J., Åkesson, L., Xu, H., Sullivan, D.P., Dai, S., Lan, J., Jinmo, P., et al. (2019). Analysis of the Human Protein Atlas image classification competition. Nat. Methods *16*, 1254–1261. https://doi.org/10.1038/s41592-019-0658-6.

14. Uhlén, M., Fagerberg, L., Hallström, B.M., Lindskog, C., Oksvold, P., Mardinoglu, A., Sivertsson, Å., Kampf, C., Sjöstedt, E., Asplund, A., et al. (2015). Tissue-based map of the human proteome. Science *347*, 1260419. https://doi.org/10.1126/science.1260419.

15. Grover, A., and Leskovec, J. (2016). node2vec: scalable feature learning for networks. In Proceedings of the 22nd ACM SIGKDD International Conference on Knowledge Discovery and Data Mining (KDD)), pp. 855–864. https://doi.org/10.1145/2939672.2939754.

16. Giurgiu, M., Reinhard, J., Brauner, B., Dunger-Kaltenbach, I., Fobo, G., Frishman, G., Montrone, C., and Ruepp, A. (2019). CORUM: the comprehensive resource of mammalian protein complexes—2019. Nucleic Acids Res. *47*, D559–D563. https://doi.org/10.1093/nar/gky973.

17. Szklarczyk, D., Morris, J.H., Cook, H., Kuhn, M., Wyder, S., Simonovic, M., Santos, A., Doncheva, N.T., Roth, A., Bork, P., et al. (2017). The STRING database in 2017: quality-controlled protein-protein association networks, made broadly accessible. Nucleic Acids Res. *45*, D362–D368. https://doi.org/10.1093/nar/gkw937.

18. Drew, K., Wallingford, J.B., and Marcotte, E.M. (2021). hu.MAP 2.0: integration of over 15,000 proteomic experiments builds a global compendium of human multiprotein assemblies. Mol. Syst. Biol. *17*, e10016. https://doi.org/10.15252/msb.202010016.

19. Schaffer, L.V., Hu, M., Qian, G., Moon, K.M., Pal, A., Soni, N., Latham, A.P., Pontano Vaites, L., Tsai, D., Mattson, N.M., et al. (2025). Multimodal cell maps as a foundation for structural and functional genomics. Nature *642*, 222–231. https://doi.org/10.1038/s41586-025-08878-3.

20. Qin, Y., Huttlin, E.L., Winsnes, C.F., Gosztyla, M.L., Wacheul, L., Kelly, M.R., Blue, S.M., Zheng, F., Chen, M., Schaffer, L.V., et al. (2021). A multi-scale map of cell structure fusing protein images and interactions. Nature *600*, 536–542. https://doi.org/10.1038/s41586-021-04115-9.

21. McInnes, L., Healy, J., and Melville, J. (2018). UMAP: uniform manifold approximation and projection for dimension reduction. Preprint at arXiv. https://doi.org/10.48550/arXiv.1802.03426.

22. Heusel, M., Bludau, I., Rosenberger, G., Hafen, R., Frank, M., Banaei-Esfahani, A., van Drogen, A., Collins, B.C., Gstaiger, M., and Aebersold, R. (2019). Complex-centric proteome profiling by SEC-SWATH-MS. Mol. Syst. Biol. *15*, e8438. https://doi.org/10.15252/msb.20188438.

23. Huang, A.C., Hsieh, T.H.S., Zhu, J., Michuda, J., Teng, A., Kim, S., Rumsey, E.M., Lam, S.K., Anigbogu, I., Wright, P., et al. (2025). X-atlas/Orion: genome-wide Perturb-seq datasets via a scalable Fix-Cryopreserve platform for training dose-dependent biological foundation models. Preprint at bioRxiv. https://doi.org/10.1101/2025.06.11.659105.

24. Jassal, B., Matthews, L., Viteri, G., Gong, C., Lorente, P., Fabregat, A., Sidiropoulos, K., Cook, J., Gillespie, M., Haw, R., et al. (2020). The Reactome pathway knowledgebase. Nucleic Acids Res. *48*, D498–D503. https://doi.org/10.1093/nar/gkz1031.

25. Alliance of Genome Resources Consortium (2020). Alliance of Genome Resources Portal: unified model organism research platform. Nucleic Acids Res. *48*, D650–D658. https://doi.org/10.1093/nar/gkz813.

26. Reicher, A., Reiniš, J., Ciobanu, M., Růžička, P., Bulanová, M., Siklos, M., Kartysh, V., Tomek, T., Koren, A., Rendeiro, A.F., and Kubicek, S. (2024). Pooled multicolour tagging for visualizing subcellular protein dynamics. Nat. Cell Biol. *26*, 745–756. https://doi.org/10.1038/s41556-024-01407-w.

27. Kahn, R.A., Virk, H., Laflamme, C., Houston, D.W., Polinski, N.K., Meijers, R., Levey, A.I., Saper, C.B., Errington, T.M., Turn, R.E., et al. (2024). Antibody characterization is critical to enhance reproducibility in biomedical research. eLife *13*, e100211. https://doi.org/10.7554/eLife.100211.

28. Serebrenik, Y.V., Mani, D., Maujean, T., Burslem, G.M., and Shalem, O. (2024). Pooled endogenous protein tagging and recruitment for systematic profiling of protein function. Cell Genom. *4*, 100651. https://doi.org/10.1016/j.xgen.2024.100651.

29. Sansbury, S.E., Serebrenik, Y.V., Lapidot, T., Smith, D.G., Burslem, G.M., and Shalem, O. (2025). Pooled tagging and hydrophobic targeting of endogenous proteins for unbiased mapping of unfolded protein responses. Mol. Cell *85*, 1868–1886.e12. https://doi.org/10.1016/j.molcel.2025.04.002.

30. Hartley, M., Kleywegt, G.J., Patwardhan, A., Sarkans, U., Swedlow, J.R., and Brazma, A. (2022). The BioImage Archive—building a home for life-sciences microscopy data. J. Mol. Biol. *434*, 167505. https://doi.org/10.1016/j.jmb.2022.167505.

31. Kohler, D., Staniak, M., Tsai, T.-H., Huang, T., Shulman, N., Bernhardt, O.M., MacLean, B.X., Nesvizhskii, A.I., Reiter, L., Sabidó, E., et al. (2023). MSstats version 4.0: statistical analyses of quantitative mass spectrometry-based proteomic experiments with chromatography-based quantification at scale. J. Proteome Res. *22*, 1466–1482. https://doi.org/10.1021/acs.jproteome.2c00834.

32. Cox, J., and Mann, M. (2008). MaxQuant enables high peptide identification rates, individualized p.p.b.-range mass accuracies and proteome-wide protein quantification. Nat. Biotechnol. *26*, 1367–1372. https://doi.org/10.1038/nbt.1511.

33. Shannon, P., Markiel, A., Ozier, O., Baliga, N.S., Wang, J.T., Ramage, D., Amin, N., Schwikowski, B., and Ideker, T. (2003). Cytoscape: a software environment for integrated models of biomolecular interaction networks. Genome Res. *13*, 2498–2504. https://doi.org/10.1101/gr.1239303.

34. Bao, F., Deng, Y., Wan, S., Shen, S.Q., Wang, B., Dai, Q., Altschuler, S.J., and Wu, L.F. (2022). Integrative spatial analysis of cell morphologies and transcriptional states with MUSE. Nat. Biotechnol. *40*, 1295. https://doi.org/10.1038/s41587-022-01498-6.

STAR★METHODS

KEY RESOURCES TABLE

REAGENT or RESOURCE	SOURCE	IDENTIFIER
Antibodies		
anti-FLAG® M2 antibody, Mouse monoclonal	Sigma F1804	AB_262044
Goat anti-mouse HRP-conjugated secondary antibody	Invitrogen 31430	AB_228307
anti-beta Tubulin antibody, rabbit polyclonal	Abcam ab15568	AB_2210952
anti-CALR antibody, chicken polyclonal	Abcam ab2908	AB_303403
Goat anti-Mouse IgG (H + L) Highly Cross-Adsorbed Secondary Antibody, Alexa Fluor™ 488	Invitrogen A11029	AB_2534088
Goat anti-Chicken IgY (H + L) Cross-Adsorbed Secondary Antibody, Alexa Fluor™ Plus 555	Invitrogen A32932	AB_2762844
Goat anti-Rabbit IgG (H + L) Cross-Adsorbed Secondary Antibody, Alexa Fluor™ 647	Invitrogen A21244	AB_2535792
Pierce anti-DYKDDDDK Magnetic Agarose beads	Thermo Scientific	A36798; AB_3106985
Bacterial and virus strains		
NEB stable	NEB C3040	N/A
Chemicals, peptides, and recombinant proteins		
DMEM, High Glucose, Pyruvate	Gibco	11995073
Fetal bovine serum, Value, US origin	Gibco	16140071
Penicillin-Streptomycin (10,000 U/mL)	Gibco	15140122
Dimethyl sulfoxide (DMSO)	Thermo Scientific	036480-AP
Trypsin-EDTA (0.05%), phenol red	Gibco	25300054
Lipofectamine 2000	Thermo Scientific	11668500
RIPA lysis and extraction	Thermo Scientific	89901
cOmplete Mini EDTA-free Protease Inhibitor Cocktail	Roche	11836153001
PhosSTOP phosphatase inhibitor cocktail	Roche	04906837001
SuperBlock Blocking Buffer	Thermo Scientific	37535
fibronectin	Sigma	F1141
4% paraformaldehyde in PBS	Thermo Scientific	19943
DAPI	Thermo Scientific	D21490
Pierce 3× DYKDDDDK FLAG peptide	Thermo Scientific	A36805
RapiGest SF Surfactant	Waters	186001861
Sequencing Grade Modified Trypsin	Promega	V511C
Critical commercial assays		
HRP chemiluminescent kit	Thermo Scientific	ZF391331
QuickExtract	Sigma	LGCQE09050
Deposited data		
Mass spectrometry proteomics data	PRIDE/ProteomeXchange	PRIDE: PXD067895
Microscopy image data	BioImage Archive	BioImage Archive: S-BIAD2261
Original code	Zenodo	Zenodo: https://doi.org/10.5281/zenodo.20548576
Development version code	GitHub	GitHub: https://github.com/idekerlab/hit_map
Experimental models: Cell lines		
HEK293T	Previously owned	CVCL_0063
3xFLAG-tagged HEK293T cell lines	This paper	N/A
9xFLAG-tagged HEK293T cell lines	This paper	N/A

(*Continued on next page*)

Continued		
REAGENT or RESOURCE	SOURCE	IDENTIFIER
Oligonucleotides		
Oligos used to clone target-gRNA spacers	This paper	N/A
Primers used for PCR validation of tag insertions	This paper	N/A
Recombinant DNA		
pDNR-3xFLAG-T2A-3xPuroR-Frame 0	Addgene	Addgene_245866
pDNR-3xFLAG-T2A-3xPuroR-Frame 1	Addgene	Addgene_245867
pDNR-3xFLAG-T2A-3xPuroR-Frame 2	Addgene	Addgene_245868
pDNR-9xFLAG-T2A-3xPuroR-Frame 0	Addgene	Addgene_245869
pDNR-9xFLAG-T2A-3xPuroR-Frame 1	Addgene	Addgene_245870
pDNR-9xFLAG-T2A-3xPuroR-Frame 2	Addgene	Addgene_245871
pCAS	Addgene	Addgene_206994
pDNR-gRNA	Addgene	Addgene_206990
Software and algorithms		
Python, version 3.8/3.9	Python Software Foundation	N/A
R version 4.2.3	R Foundation for Statistical Computing	N/A
MSstats Version 4.8.3	MSstats R/Bioconductor package[31]	N/A
MaxQuant Version 1.6.12.0	Open source[32]	N/A
Cytoscape Version 3.10.4 Beta1	Cytoscape Consortium[33]	N/A
Other		
96-well glass-bottom plates	MatTek	P96G-1.5-5-F
Keyence BZ-X800 microscope	Keyence	BZ-X800
Nikon CFI60 Plan Apo λD 60× oil objective	Nikon	MRD71670
PepSep column, 15 cm × 150 μm ID, 1.5 μm BEH	Bruker	1893474
BioPureSPE Mini 96-well plate	The Nest Group	20 mg PROTO 300 C18
KingFisher Flex Purification System	Thermo Fisher Scientific	N/A
Vanquish *Neo* HPLC platform	Thermo Fisher Scientific	N/A
Exploris 480 Orbitrap Mass Spectrometer	Thermo Fisher Scientific	N/A

EXPERIMENTAL MODEL AND STUDY PARTICIPANT DETAILS

Plasmid construction for endogenous protein tagging

Plasmids were derived from a previous study.[11] The tag components were modified to include either 3xFLAG or 9xFLAG (Table S18). Target-gRNAs were designed by selecting spacer sequences that bind to the most C-terminal end of each gene from the CRISPick results for the library. All the spacer oligos were cloned into a CROP-seq compatible vector with blasticidin resistance (Table S19).

Generation of tagged HEK293T cell lines

HEK293T cells were cultured in DMEM media supplemented with 10% FBS and Penicillin-Streptomycin. Given the size of the pilot library a simplified tagging approach was used based on the HITAG reagents. HEK293T cells were plated on 24-well plates at 50% confluency per well. A plasmid mixture consisting of 100 ng pCas, 50 ng target-gRNA, 50 ng pDNR-gRNA, and 500 ng corresponding pDNR was transfected using Lipofectamine 2000. Post transfection, cells were passaged 1:4 whenever confluent and plated into non-drug containing media. On the seventh day after transfection, the cells were split 1:4 into media supplemented with puromycin 0.5 μg/mL. The selection process continued until all control (non-tagged) cells in a parallel well had died under puromycin selection.

Tagged cell lines were validated by analyzing the junction sequences between the target gene and the tag with genomic DNA extracted using the Lucigen QuickExtract reagent. Junction regions were PCR amplified with one primer binding to the tag and the other primer binding to the target gene (Figure S4, Table S2). The amplified bands were sent for Sanger sequencing to validate that the tagging had occurred as anticipated and that the tags were in-frame with the upstream gene of interest (Figure S5).

Cryostocks were generated either in 2 mL cryovials or on 24-well plates. For cryovial stocks, trypsinized cells were resuspended in culture media supplemented with 10% DMSO. These cell suspensions were then frozen using a Mr. Frosty freezing container. For a 24-well plate, 250 μL of trypsin solution was added to each well and the plate was incubated at 37°C for 5 min. An equal volume of media with 40% FBS containing 20% DMSO was then added to each well. The plates were sealed with aluminum foil and wrapped with parafilm on the outside. Plates were then placed in a styrofoam container and the entire container was moved into −80°C freezer for >24 h before being removed from the container and stored at −80°C or in liquid nitrogen longterm.

METHOD DETAILS

Western blot analysis

The expression of the tagged proteins was validated through western blot. Tagged cell lines were expanded in 6-well plates with puromycin containing media. Cells at approximately 80% confluency were harvested using ice-cooled RIPA buffer (R0278) supplemented with a proteinase inhibitor cocktail (Merck 11836153001). The cell lysates were incubated on ice for 20 min and then centrifuged at 13,000 rpm for 10 min. Protein concentrations in the supernatant were normalized by further diluting samples as needed with a lysis buffer. Samples were prepared using an LDS loading buffer, followed by boiling at 95°C for 5 min. Protein samples (Figure S1, 21.5 μg proteins per lane; Figure S2, 10.5 μg proteins per lane) were loaded onto Bis-Tris 4-12% gels. Proteins were separated by electrophoresis and transferred to PVDF membranes using a quick transfer system (BioRad 1704156).

The membrane was blocked with Superblock (Thermo 37535) for 1 h at room temperature, then incubated overnight at 4°C with anti-FLAG primary antibody (Sigma F1804, 1:1000) in Superblock with 0.1% Tween 20. After three washes with TBS-T buffer (TBS with 0.1% TWEEN 20), the membrane was incubated with anti-mouse HRP-conjugated secondary antibody (Invitrogen 31430, 1:100,000) in TBS-T buffer for 1 h at room temperature. Membranes were then washed 3 more times with TBS-T buffer. Protein bands were visualized using an HRP chemiluminescent kit (Thermo ZF391331) with optimized exposure times. Image background subtraction was performed using the ImageJ Subtract Background function (rolling ball radius: 50 pixels, light background, sliding paraboloid). Contrast and brightness were adjusted uniformly for clarity, and images were at times flipped along their vertical axis to simplify their presentation in figures. All processing parameters were applied equally across all images.

Acquiring IF images

Fixed cell samples for immunofluorescence imaging were prepared on 96-well glass bottom plates (MatTek, P96G-1.5-5-F) coated with fibronectin. Fibronectin was 5 times diluted in DDW from the commercial product (Sigma F1141). Cells were fixed using 4% paraformaldehyde in PBS for 15 min at room temperature. After two washes with PBS containing 0.1% Triton X-100, cells were blocked with Superblock supplemented with 0.1% Triton X-100 for 2 h at room temperature. Then cells were treated with Superblock containing 0.1% Triton X-100 and primary antibodies, including anti-β-tubulin (Abcam, ab15568, 1:500), anti-CALR (Abcam, ab2908, 1:500), and anti-FLAG (Sigma F1804, 1:500) for 2 h at room temperature. After three washes with PBS, the samples were incubated with fluorescent secondary antibodies (Invitrogen A11029, 1:1000; Invitrogen A32932, 1:1000; Invitrogen A21244, 1:1000) in Superblock containing 0.1% Triton X-100 for 1 h, followed by 15 min of DAPI staining and a final wash with PBS. PBS with 75% glycerol was added to preserve the samples.

Immunofluorescence images were captured at the University of California, San Diego, utilizing either a confocal microscope (Leica SP8, UCSD neuroscience microscopy core) or a wide-field microscope (Keyence BZ-X800, Lens: Nixon MRD71670, CFI60 PlanApo λD 60× oil, N.A. 1.42, W.D. 0.15mm, F.O.V. 25mm). We captured immunofluorescence images of the tagged protein of interest (Alexa Fluor 488) and three cellular landmarks: nucleus (DAPI), endoplasmic reticulum (Alexa Fluor 555), and microtubules (Alexa Fluor 647). To capture accurate 3D cell structures, z stack images were acquired with 31 frames at 0.3 μm intervals over a 9 μm range. The z stack images captured from confocal microscopes were combined into one maximum intensity projection. Unlike confocal microscopes, wide-field systems capture both in-focus and out-of-focus light, which can obscure the signal. To address this, we applied Richardson-Lucy deconvolution with a theoretical point-spread function (PSF), iteratively refining each layer and projecting the Z-stacks to achieve confocal-like image quality.[12] These deconvolved images were then used as input for a pre-trained deep learning model to generate embeddings that preserve protein localization information.[13]

AP-MS sample preparation

For affinity purification, all 16 tagged lines in the HEK293T background (both 3xFLAG and 9xFLAG-tagged lines), were grown in 15-cm dishes to ∼80% confluency under puromycin selection media (DMEM+10%FBS+1% PenStrep+ 0.5 μg/mL puromycin). Cell pellets were generated by washing, scraping, and collecting cells in ice-cold Dulbecco's PBS. Parental HEK293T cells were used as controls for all the experiments. Four biological replicates were processed per tagged line, including control conditions. Cell pellets were lysed with 650 μL of ice-cold IP lysis buffer containing 0.1% NP-40, 0.1% Tween 20, 10% glycerol, 100 mM KCl, 5 mM MgCl2, 20 mM Tris-HCl pH 8.0, 1× protease and phosphatase inhibitor cocktail tablets [cOmplete Mini EDTA-free protease and PhosSTOP phosphatase inhibitor cocktails (Roche)] and sonicated 3 times at 7% power for 10 s each before centrifugation at 13,000 × g for 30 min at 4°C. For each affinity purification, 1mL of lysate normalized to 2 mg total protein was processed on a KingFisher Flex (KFF) Purification System (Thermo Scientific) at 4°C. Briefly, 20 μL of Pierce Anti-DYKDDDDK Magnetic Agarose beads (Thermo Scientific, A36798) was incubated with 1.0 mL cell lysate for 2 h and protein-bound beads were washed three times

with 1.0 mL IP lysis Buffer and then once with 1.0 mL IP buffer containing 50 mM Tris–HCl, pH 7.4 at 4°C, 150 mM NaCl, 1 mM EDTA. Bound proteins were eluted twice with 50 μL of 0.1 mg/mL 3xFLAG peptide, 0.05% RapiGest SF (Waters, 186001861) in IP Buffer for 30 min each at 23°C.

Protein digestion and peptide clean up

For digestion, 50μL FLAG-AP eluate was denatured at 37°C for 30 min in 50 mM Tris–HCl pH 8.0, 2 M urea buffer complemented with 1 mM DTT. Samples were then alkylated at room temperature in the dark for 45 min by addition of 3 mM iodoacetamide, followed by quenching for 10 min with 3 mM DTT. Trypsin (1.5 μg; Promega) was added and samples were incubated overnight at 37°C with shaking at 1,000 rpm. Peptides were acidified with TFA to a final concentration of 0.5%, pH < 2.0, desalted at room temperature using a BioPureSPE Mini 96-well plate (20 mg PROTO 300 C18; The Nest Group) as per manufacturer instruction and dried under vacuum centrifugation using a CentriVap Concentrator (Labconco).

LC-MS/MS acquisition

For LC-MS/MS acquisition, dried samples were resuspended in 30 μL of 0.1% formic acid before filtering through 0.45-μm filter and injection (2 μL) onto the Thermo Scientific Vanquish *Neo* HPLC platform on-line with a Thermo Exploris 480 Orbitrap Mass Spectrometer. Peptides were separated using a Bruker 15-cm-long 150-um ID PepSep column packed with 1.5-μm BEH particles, over a 45-min gradient with mobile phase A composed of 0.1% formic acid in water and mobile phase B composed of 0.1% formic acid in 80% acetonitrile. The chromatographic gradient was run at a flow rate of 600 nL/min throughout. The gradient started at 4% B before increasing to 28% B over 30 min, followed by an increase to 45% B over 5 min, and finally finishing with a wash of 95% B for 9 min. Full scans were collected at a resolution of 120,000, 350–1,250 m/z scan range with a normalized AGC target set to 100% and the maximum injection time set to "Auto". Data-dependent scans were collected at a resolution of 15,000 with a normalized AGC target of 200% and maximum injection time set to "Auto". Precursors were selected for sequencing based on an allowed charge state of 2–6, and a dynamic exclusion after two sequencing events for 20 s of precursors within 10 ppm. The total cycle time of the full MS scan and all dependent MS2 scans was 1 s.

MS database search and protein quantification

Raw MS data were searched against the UniProt canonical isoforms of the human proteome, downloaded April 4, 2023, using the default settings in MaxQuant version 1.6.12.0, with match-between-runs enabled.[32] Peptides and proteins were filtered to 1% FDR in MaxQuant, and identified proteins were then subjected to PPI scoring.

AP-MS peptide level data from MaxQuant were summarized to protein level data using the R package MSstats version 4.8.3,[31] with the dataProcess function with default settings except featureSubset = "highQuality" and remove_uninformative_feature_outer = TRUE. The matrix (protein rows x MS-run columns) of log transformed protein intensities were then subjected to another round of normalization by subtracting the median polish column effects (R function medpolish) from corresponding columns. AP-MS runs for bait TOMM40 were excluded from all further analysis because the observed intensity for TOMM40 was only a small fraction (16%) of that observed in control runs. The mass spectrometry proteomics data have been deposited to the ProteomeXchange Consortium via the PRIDE partner repository with the dataset identifier PXD067895.

Data processing: Wide field image deconvolution

We performed deconvolution of wide field fluorescence microscopy images using deconwolf (https://github.com/elgw/deconwolf), an open-source and GPU-accelerated tool that offers high-speed, high-fidelity 3D deconvolution. We generated a theoretical, system-specific point-spread function (PSF). The PSF was computed via the Born–Wolf model, which integrates optical physics over sensor pixels to capture precise diffraction behavior (refractive index: 1.518, numerical aperture: 1.4, lateral resolution: 130nm and axial resolution: 300nm). Deconvolution was carried out using the Richardson–Lucy algorithm accelerated by a scaled heavy-ball optimization and is compatible with GPU processing for substantial speed gains for 100 iterations. The deconvolved images for each channel of each protein are z-max projected for stacking. By comparing the correlation of the embedding of the deconvolved images with that of the reference HPA images, we scanned a range of regularization parameter (psigma) controlling smoothing and noise suppression to optimize it with 5-fold cross-validation. To improve local contrast in fluorescence microscopy images, we applied Contrast Limited Adaptive Histogram Equalization (CLAHE) using OpenCV. CLAHE adaptively equalizes contrast within small image tiles while limiting noise amplification in homogeneous regions. Grayscale images were processed with a tile grid size of 8 $\times$ 8 pixels, and the clip limit (saturation level) was set to 0.7.

Data processing: AP-MS PPI extraction

Bait-prey interactions were scored using a custom computational pipeline developed in-house using R version 4.2.3 for high-confidence PPI mapping of endogenously tagged proteins coupled with AP-MS. Briefly, the algorithm incorporates a modified *Z* score labeled as ZMADEX, which uses median absolute deviations (MAD) and excludes (EX) any values from the scored bait and related baits while calculating the median and MAD. More precisely, ZMADEX for a prey K with intensity X in bait J is given by the formula ZMADEX = (X - median_ex_x)/MAD_ex_x, where median_ex_x and MAD_ex_x are median and MAD, respectively, calculated on the distribution of intensities for the prey K excluding values for bait J and related baits of J. The ZMADEX scores were calculated per

CellPress
OPEN ACCESS

replicate MS-run, and the means across all replicates, with missing values set to zero, were used as the final scores per bait-prey. Mean ZMADEX scores $\geq$3 were accepted as true interactors, provided that they also passed filter 2, described below.

For comparison with control conditions (filter 2), we used the MSstats function "groupComparison" to perform t-tests comparing per-bait means of log-transformed intensities, using all groups to calculate a pooled variance. Any significant increases over control ($p \leq 0.01$) were accepted, provided that they also pass the ZMADEX filter described above. Full details of the pipeline will be described in a separate publication.

Gold standard interactor and decoy sets for ROC analysis were taken from the STRINGDB human subset (v12.0, downloaded 11/12/2024) and CORUM (v5.0, downloaded 10/31/2024). The STRINGDB interactors comprised all pairs of MS-observed proteins (identified by MaxQuant in our data) with direct physical links that included a bait protein with combined score >0.6. The CORUM true interactors comprised all pairs of MS-observed proteins that included a bait protein that co-occurred as members of at least one complex. The decoys comprised all bait-prey pairs of MS-observed proteins within the STRINGDB physical edge subnetwork with combined score >0.6 that were not within three or fewer steps from a bait. False positive rate was calculated as the number of decoys that passed a threshold divided by the total number of decoys. True positive rate was calculated as the number of CORUM and/or STRING gold standard interactors divided by the size of the union of STRINGDB and CORUM gold standard sets. The 9× and 3× AP-MS datasets were analyzed separately for all steps above.

PPI network visualization was performed using Cytoscape version 3.10.4-BETA1.[33] Detailed MSacquisition and MaxQuant search parameters are provided (Table S20).

Data embedding

We selected the intersection of the IF and high-quality AP-MS interactors (ZMADEX scores $\geq$ 3), resulting in 14/16 proteins. Proteins were first embedded within each modality separately, as described previously.[20] These embeddings were combined with self-supervised machine learning to build a unified multimodal embedding.[19] The deconvolved images are embedded using the DenseNet-121, a convolutional neural network optimized for classification of protein localization.[13] Feeding the deconvolved 4-channel images into this model, we obtained an embedding of 1024-dimension for each protein. The node2vec Python3 implementation (https://github.com/eliorc/node2vec) was used to encode each protein as a 1024-dimension feature vector based on its protein-protein interaction neighborhood ($p = 1$, $q = 1$, walk length = 8, number of walks = 15). The unified embedding is generated from a self-supervised trained model with autoencoder structure based on multimodal structured embedding.[19,34] The loss function combines a reconstruction loss and triplet loss: the reconstruction loss preserves information from the original modalities, and the triplet loss merges the two modalities together.

For validation of CCDC12 association with spliceosome, the HEK293T perturb-seq data was collected from X-Atlas/Orion dataset.[23] Preprocessed scRNA-seq dataset containing only valid guides were directly downloaded from https://doi.org/10.25452/figshare.plus.29190726.v1. We then processed the data as described in the original analysis.[23] For this analysis, we first identified expressed genes in the dataset by: (1) converting the counts to cp10k (counts per 10,000) by normalizing the total counts; (2) for each gene, calculating the mean expression value in cells with non-targeting guides; (3) defining the expressed genes as those whose mean expression collectively account for 99% of the total. Next, we conducted a batch-wise normalization against the control to quantify the expression changes. This included: (1) calculating gene expression mean and standard deviation in control cells, for each gene in each batch; (2) normalizing all gene expression with their corresponding mean and standard deviation in the control cells in each batch; (3) aggregating cells across batches to retrieve a pseudobulk profile for each perturbation. We select the top 500 most variable genes of all the perturbations to select the perturbation with the most similar profiles as that of the CCDC12.

Calculating costs of conventional approaches vs. HIT-MAP

The total costs of the conventional approach and the HIT-MAP pipeline are determined by several factors, including materials and equipment costs (Tables S13–S15). For our conventional approaches, we selected the methods which were used to generate the current large IF and AP-MS datasets.[1–3,14] Labor costs vary substantially depending on the complexity of cell line generation, the need to develop and validate target-specific antibodies, and the level of technical skill available. Instrument costs also fluctuate with access to equipment and operational logistics. Although the HIT-MAP pipeline potentially streamlines experimental workflows—reducing manual effort and simplifying required instrumentation—these advantages are difficult to quantify in a standardized manner. To ensure a fair and transparent comparison, we focused primarily on direct material costs with a comparative overview of operational logistics and throughput (or labor intensity) to illustrate the broader scalability of the HIT-MAP pipeline. Furthermore, while HIT-MAP benefits because the same cell lines used for IF can also be used for AP-MS, we do not factor in that cost savings.

A key distinction between the two methods is that the HIT-MAP pipeline requires an additional preparatory step for generating clonal cell lines: construction of gRNA plasmid libraries required for endogenous tagging; generation of a gRNA-expressing cell pool; delivery of donor tagging constructs and selection of successfully tagged populations; and single-cell sorting followed by clonal expansion and validation. These preparation costs form the baseline for the HIT-MAP pipeline's cost calculations, enabling a fair comparison with the conventional method. Notably, when multiple downstream pipelines (e.g., imaging and AP/MS) are applied in parallel, these preparation costs are shared and not repeated, thereby further reducing the per-target cost. All other downstream processing steps and reagents not specifically mentioned were considered common to both methods and were therefore excluded from the cost comparison.

For imaging analysis, we compared only the costs of antibodies specific to each protein target. The average cost of a target-specific primary antibody was set as the product with the smallest volume, based on market averages. For HIT-MAP pipeline, this cost was replaced with the expense of an anti-FLAG M2 antibody (~$300 for a 100 μL product), assuming that 1 μL per sample was sufficient for staining. This was added to the previously calculated cost of generating the tagged lines.

For AP/MS analysis, the conventional method included the cost of generating stable cell lines with an overexpressed gene-of-interest tagged with an affinity tag and expanding cell numbers for sample preparation. For the HIT-MAP pipeline, we incorporated the cost of generating the endogenously tagged cell lines, as described above, plus the cost of expanding the cells for AP/MS. Because the tagged proteins are expressed endogenously, larger numbers of cells were expanded to achieve the same analytical sensitivity.

QUANTIFICATION AND STATISTICAL ANALYSIS

Statistical tests and significance

Paired comparisons of the same protein under different conditions (3×FLAG vs. 9×FLAG; raw vs. deconvolved wide-field; confocal vs. raw vs. deconvolved) were assessed by two-sided paired Student's *t* test. Independent-group comparisons of protein pairs or genes (within-vs. across-compartment IF embedding similarity, Figure 2F; shortest-path-length distributions of known, novel, and random PPI pairs, Figures S7B and S7C; Perturb-seq similarity scores; Figure 4E) were assessed by two-sided Mann–Whitney U test, which makes no normality assumption. Pearson correlation compared cross-modality distance matrices (Figure 4B). Bait–prey same-compartment enrichment (Figure S7H) and CORUM-complex odds ratios (Figure 3D) were assessed by two-sided Student's *t* test. AUROC benchmarked AP-MS PPI recovery against CORUM, STRING (combined score >0.6), and hu.MAP 2.0 (Figure 3B). Significance was defined as $p < 0.05$ (two-sided); exact *p*-values are reported throughout, and no multiple-testing correction was applied to the planned pairwise comparisons. Given $n = 16$ proteins, formal normality testing was not performed; key trends were cross-validated with non-parametric tests where the underlying distribution was uncertain.

Values of n and plotting conventions

The pilot dataset comprises 16 proteins spanning three subcellular compartments (nucleus, cytosol, mitochondria), each tagged with 3×FLAG and 9×FLAG (32 endogenously tagged HEK293T cell lines). Where n refers to proteins (e.g., Figures 2B and 2C, Figure S6C), $n = 16$. For protein-pair analyses, n refers to unordered pairs: Figure 2F, $n = 36$ within-compartment, 84 across-compartment; Figure S7B, $n = 326$ known, 193 novel, 193 random; Figure S7C, $n = 293$ known, 114 novel, 114 random. For Perturb-seq (Figure 4E), $n = 7$ (Bact stage), 5 (NTC core), 7 (A complex), 6 (E complex), 500 (random). The high-confidence PPI network (Figures 3C and 3E) comprises 576 interactions among 331 proteins from the 9×FLAG dataset; 14/16 proteins had both IF and high-confidence AP-MS data and were included in the multimodal co-embedding. In violin and boxplots, the central white dot indicates the median, the box marks the interquartile range (Q1–Q3), and red lines mark the data extent within 1.5× IQR. Confidence intervals were not computed.

Significance criteria, stratification, and sample size

High-confidence PPIs were defined as mean ZMADEX ≥3 across replicates AND MSstats groupComparison *t* test $p \leq 0.01$ versus parental HEK293T control, applied independently to the 3×FLAG and 9×FLAG datasets. Cosine similarity >0.6 defined "proximal" pairs for the STRING-distance analysis (Figure 4C). The 16 pilot proteins were selected by stratified sampling across three subcellular compartments to evaluate the pipeline across diverse localization patterns; this is a methodological pilot rather than a hypothesis-testing experiment with treatment groups, so conventional randomization was not applicable. No formal sample-size calculation was performed; $n = 16$ reflects the practical scale of an arrayed pilot, and the limitations of this scale are addressed in the Limitations of the study section. Random reference sets (Figures S7B, S7C, and S7H; Figure 4E) were drawn by uniform sampling from the relevant background pool with size matched to the comparator group where applicable.

Cell Reports Methods, Volume *6*

Supplemental information

HIT-MAP: A scalable approach to multimodal mapping of subcellular organization

Gege Qian, Joonwon Kim, Richa Tiwari, Benjamin J. Polacco, Antoine Forget, Leah V. Schaffer, Hark Kyun Kim, Jiahao Gao, Yuan Zhou, Gwendolyn M. Jang, Marcus R. Kelly, Xiaoyu Zhao, Helene Foussard, Liudeng Zhang, Nevan Krogan, Trey Ideker, and Alejandro Chavez

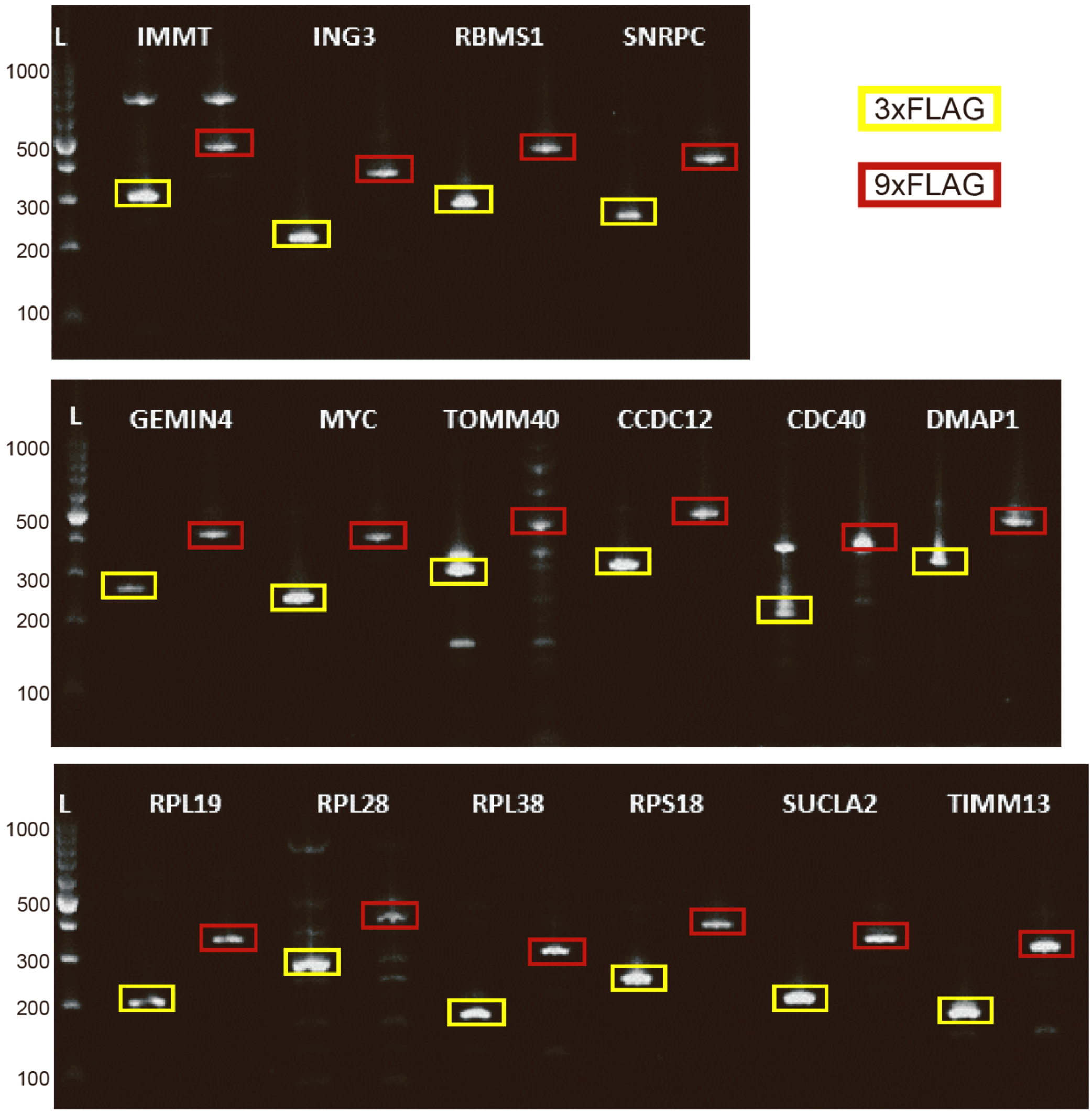

Figure S1. PCR validation for all tagged cell lines.

Knock-in is validated using PCR with one forward primer binding to the target gene and the reverse primer binding to the T2A sequence downstream of the FLAG tag. This approach ensures that PCR products are generated only when the tag is inserted in the correct orientation at the C-terminus of each target gene. The 9×FLAG construct shows larger PCR products due to the additional 6 FLAG repeats compared to the single FLAG tag.

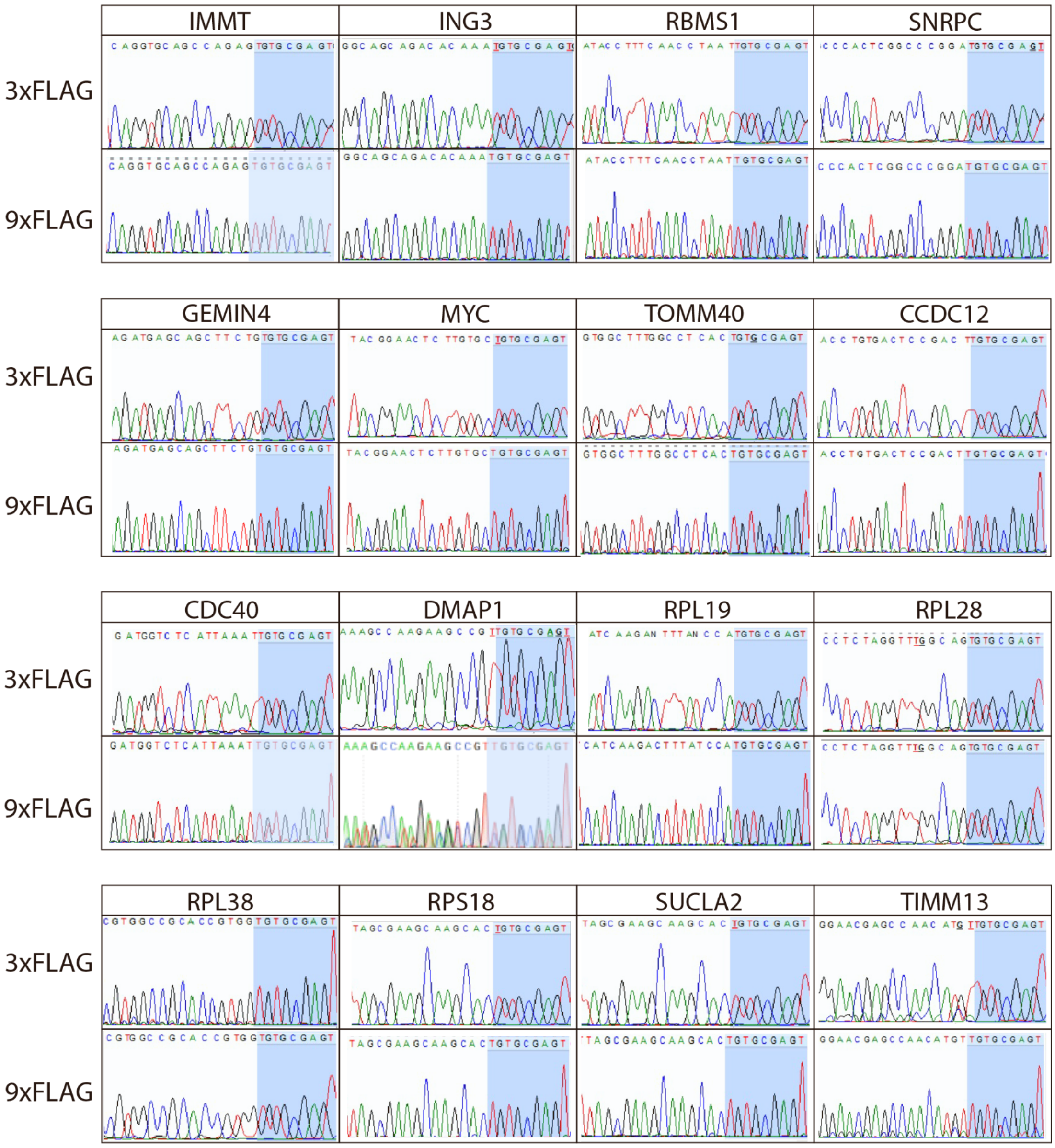

Figure S2. Sanger sequencing validation.
Sequencing analysis of PCR products confirms successful tag integration at the C-terminus of each gene. The target gene sequence (5' to 3') appears on the left, and the blue-colored section indicates where the linker sequence begins.

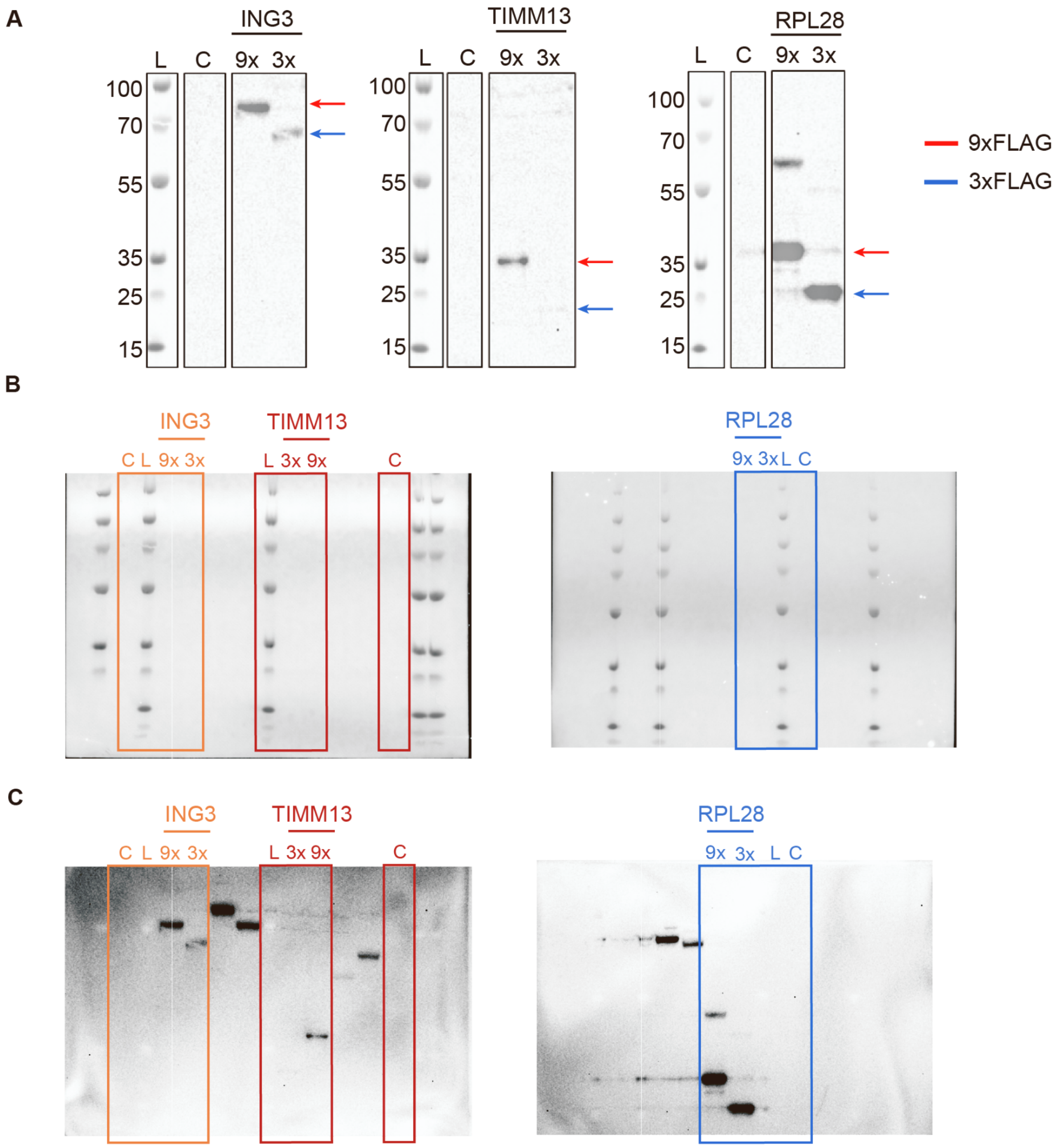

Figure S3. Western blot validation of tagging analysis.

a) Western blot analysis demonstrating the fusion of either a 9xFLAG- or 3xFLAG-tag to ING3, TIMM13, and RPL28. Red arrows indicate the 9xFLAG-tagged variant of the proteins and the black arrows indicate the 3xFLAG-tagged versions. Proteins are detected using an anti-FLAG antibody. The 9xFLAG-tagged proteins consistently showed stronger signal detection and higher molecular weight compared to their 3xFLAG-tagged counterparts (9xFLAG tag 10kDa, 3xFLAG tag 4kDa). L: ladder. C: Control untagged sample. 9x: 9xFLAG-tagged protein samples. 3x: 3xFLAG-tagged protein samples. Original image of western blot with b) colorimetric and c) chemiluminescence detection. The images are edited using imageJ to remove background noise and adjusted uniformly along all the images for clarity.

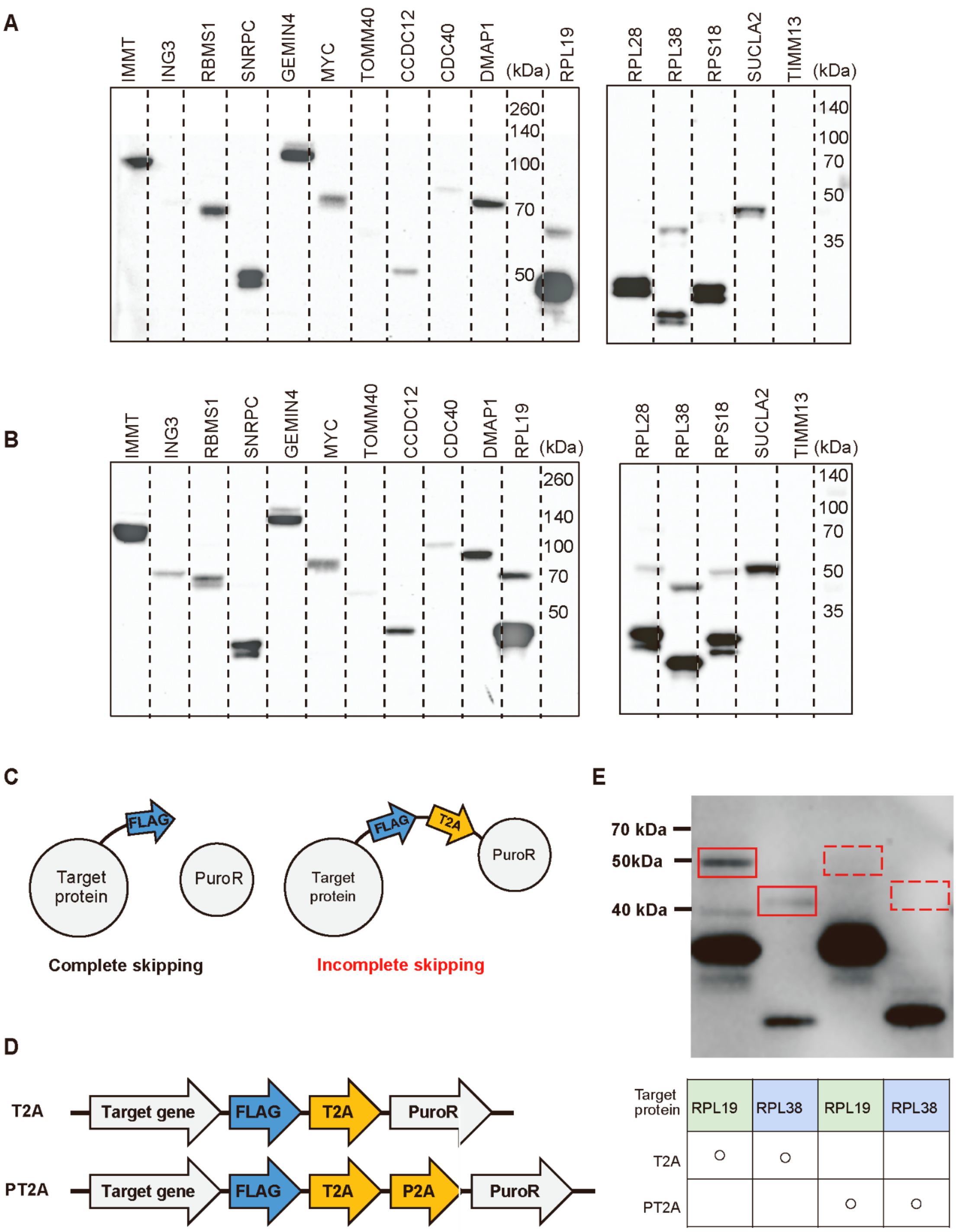

Figure S4. Western blot validation for all tagged cell lines.
Western blot validation of a) 3xFLAG-tagged cell lines and b) 9xFLAG-tagged cell lines. Lane labels indicate the name of the tagged gene in each lane. Both blots are generated using equal protein input amounts and equivalent exposure times. c) Higher molecular weight bands show in 9xFLAG-tagged proteins (e.g. RPL28) represent minor populations of uncleaved T2A-PuroR fusion products, which are more detectable due to enhanced antibody binding to the 9xFLAG tag compared to that of 3xFLAG tag. d,e) Further optimization using tandem skipping peptides (PT2A) effectively resolves incomplete cleavage, eliminating the high molecular weight fusion bands.

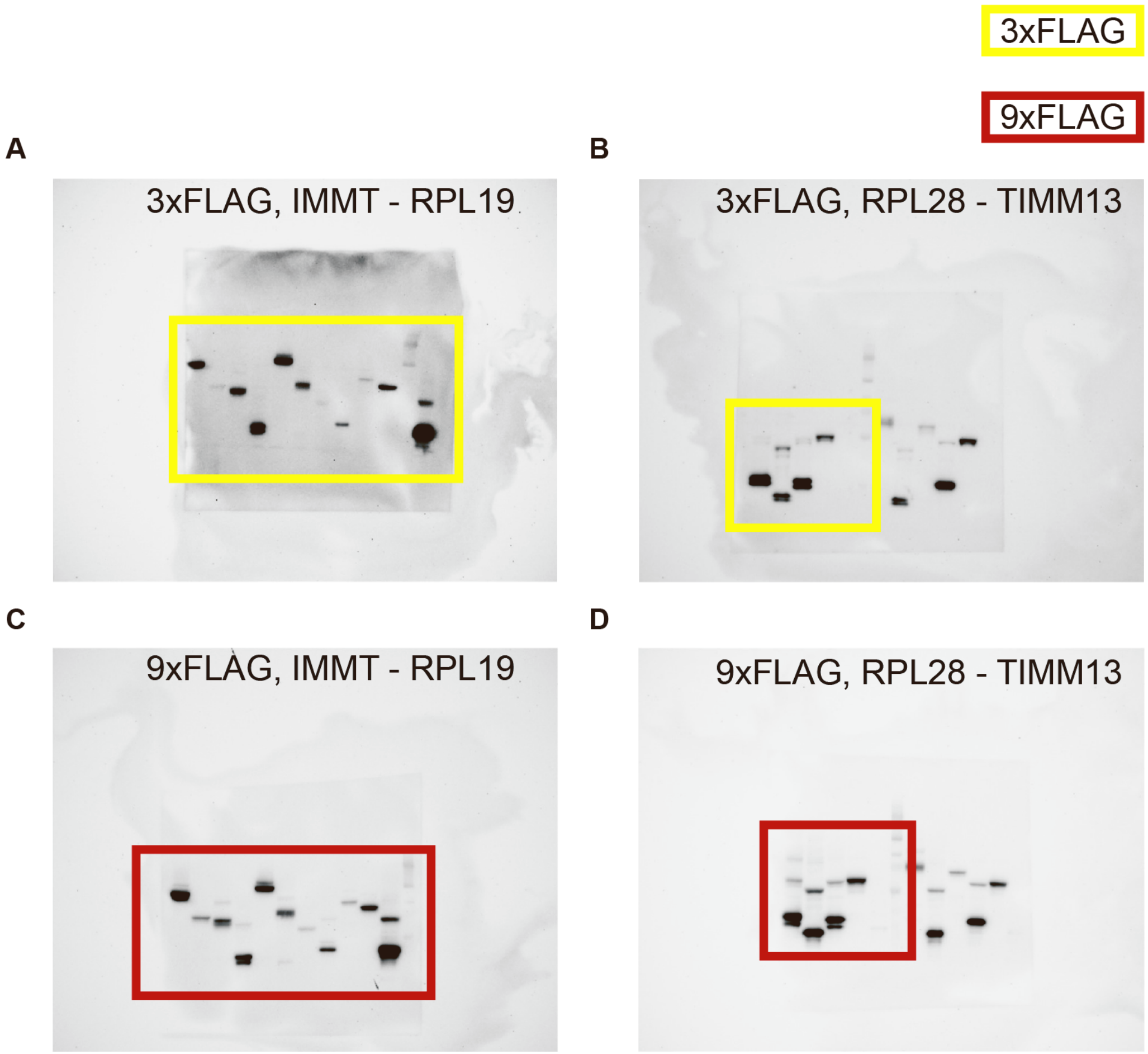

Figure S5. Raw image of Western blot validation for all tagged cell lines. a-b) 3xFLAG-tagged cell lines and c-d) 9xFLAG-tagged cell lines. The area inside the box indicates the region that has been presented in Supplemental Figure 4.

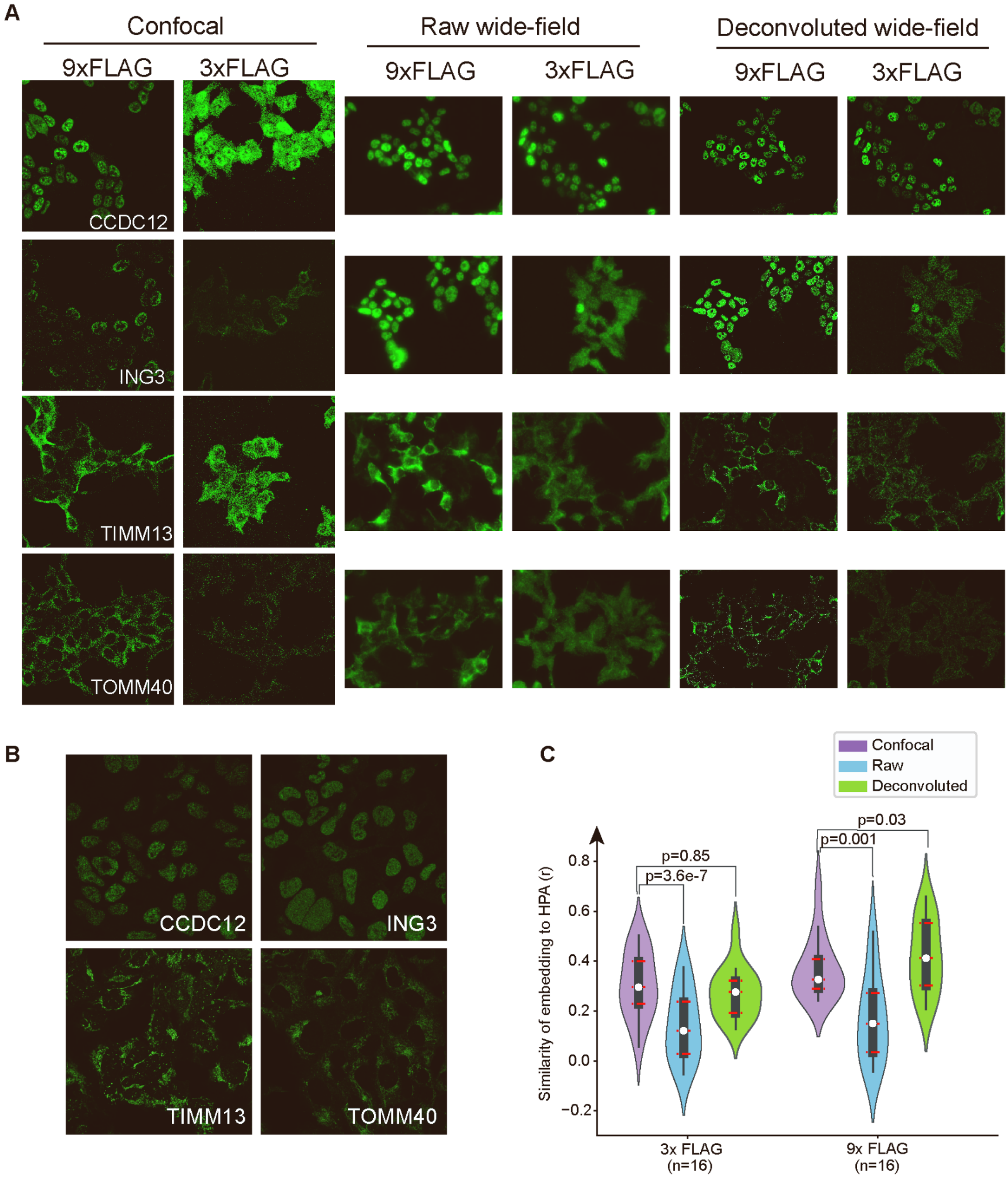

Figure S6. Tagging system validation with in-house confocal images

a) Example confocal, raw wide-field and deconvolved wide-field images of 3xFLAG and 9xFLAG tagged cell lines of CCDC12, ING3, TIMM13 and TOMM40. b) Confocal image from HPA.

c) Distribution of Pearson correlations between embeddings of different imaging platforms and image processing approaches to that of HPA for all 16 proteins tagged with either 3xFLAG or 9xFLAG.

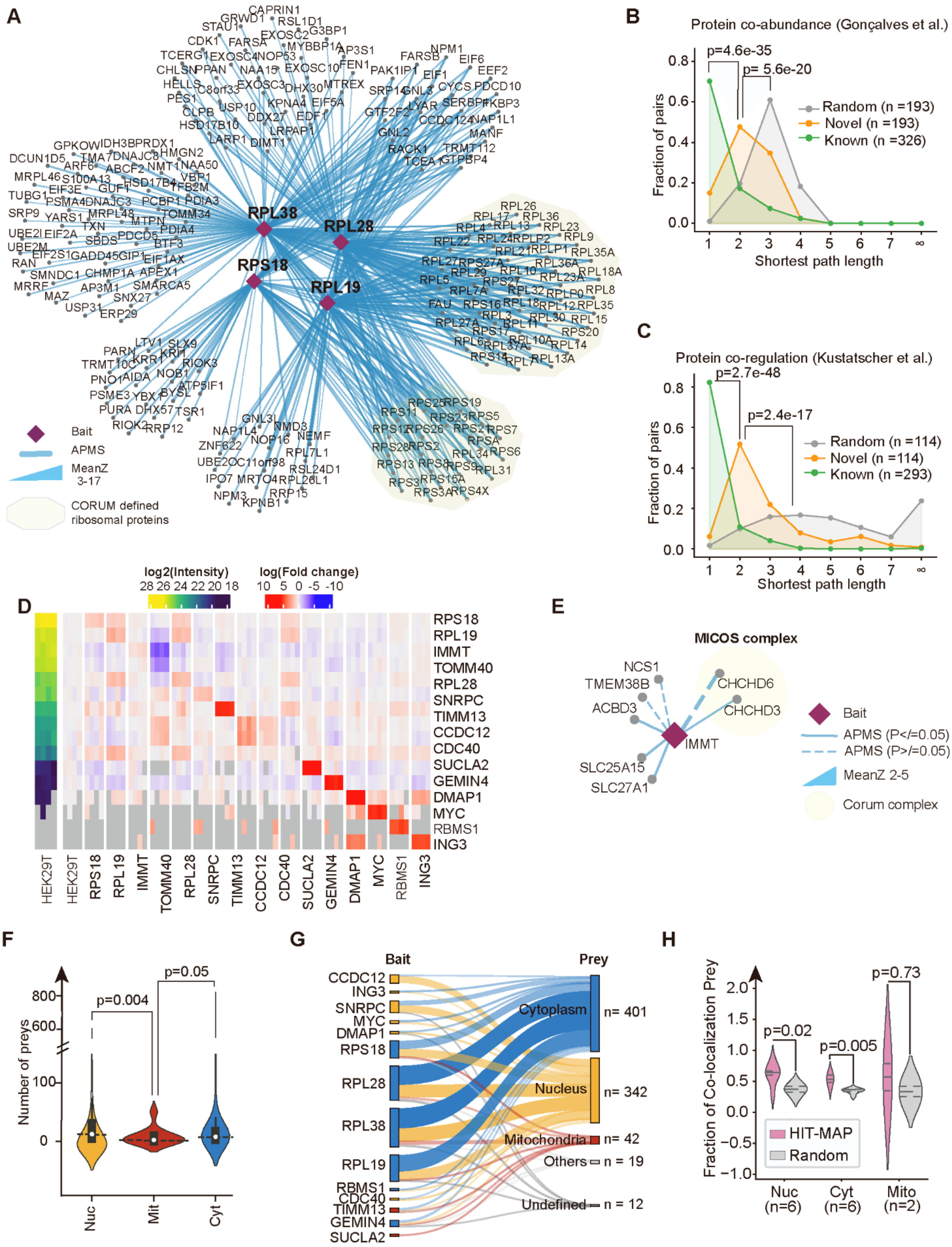
A
RPL38
RPL28
RPS18
RPL19
Bait
APMS
MeanZ 3-17
CORUM defined ribosomal proteins
B
Protein co-abundance (Gonçalves et al.)
p=4.6e-35
p= 5.6e-20
Random (n =193)
Novel (n =193)
Known (n =326)
Fraction of pairs
Shortest path length
C
Protein co-regulation (Kustatscher et al.)
p=2.7e-48
p=2.4e-17
Random (n =114)
Novel (n =114)
Known (n =293)
Fraction of pairs
Shortest path length
D
log2(Intensity)
28 26 24 22 20 18
log(Fold change)
10 5 0 -5 -10
RPS18
RPL19
IMMT
TOMM40
RPL28
SNRPC
TIMM13
CCDC12
CDC40
SUCLA2
GEMIN4
DMAP1
MYC
RBMS1
ING3
HEK29T
HEK29T
RPS18
RPL19
IMMT
TOMM40
RPL28
SNRPC
TIMM13
CCDC12
CDC40
SUCLA2
GEMIN4
DMAP1
MYC
RBMS1
ING3
E
MICOS complex
NCS1
TMEM38B
ACBD3
CHCHD6
CHCHD3
IMMT
SLC25A15
SLC27A1
Bait
APMS (P</=0.05)
APMS (P>/=0.05)
MeanZ 2-5
Corum complex
F
Number of preys
p=0.004
p=0.05
Nuc
Mit
Cyt
G
Bait
Prey
CCDC12
ING3
SNRPC
MYC
DMAP1
RPS18
RPL28
RPL38
RPL19
RBMS1
CDC40
TIMM13
GEMIN4
SUCLA2
Cytoplasm
n= 401
Nucleus
n= 342
Mitochondria
n= 42
Others
n = 19
Undefined
n = 12
H
Fraction of Co-localization Prey
p=0.02
p=0.005
p=0.73
HIT-MAP
Random
Nuc (n=6)
Cyt (n=6)
Mito (n=2)

Figure S7. AP-MS PPI analysis.

a) AP-MS detected PPI full network for ribosomal proteins, highlighting CORUM complex defined ribosomal preys. For PPI mapping, a cut-off of Mean $Z_{MADEX} \geq 3$ and $p \leq 0.01$ was used. b) Distribution of the shortest distances of the PPIs identified from AP-MS grouped into Known (included in STRING and CORUM), Novel (not documented) and Random selected set of proteins in the high confidence graph generated from protein co-abundance data (Goncalves et al.) and c) protein co-regulation data (Kustatscher et al.) d) Heatmap of mass-spectrometry intensity for all baits. The left most column shows the intensity of the negative control of HEK293T cells with no protein tagged. Red/blue heatmap shows the log2fold change of each bait compared to HEK293T control. Note: RBMS1 and ING3 were not detected in the negative control. Their fold change is their intensity divided by the intensity of MYC in the HEK293T control. e) AP-MS detected PPI network for IMMT with a relaxed threshold ($Z_{MADEX} \geq 2$). CORUM defined preys are highlighted. f) Violin plot showing the number of preys for baits annotated by their subcellular localization in OpenCell dataset (https://opencell.sf.czbiohub.org/download). Asterisks(*) indicate significantly more preys than the mean of mitochondrial baits ($p < 0.05$). g) Sankey plot with the flow representing the interaction of a bait with preys assigned to specific compartments, illustrating the distribution of interaction partners across different cellular localizations. h) Violin plot showing the percentage of prey proteins localized to the same subcellular compartment as their corresponding bait. Distributions are compared to a randomized control in which prey are randomly assigned to baits. Statistical significance was assessed using pairwise Student's t-tests, and p-values are indicated.

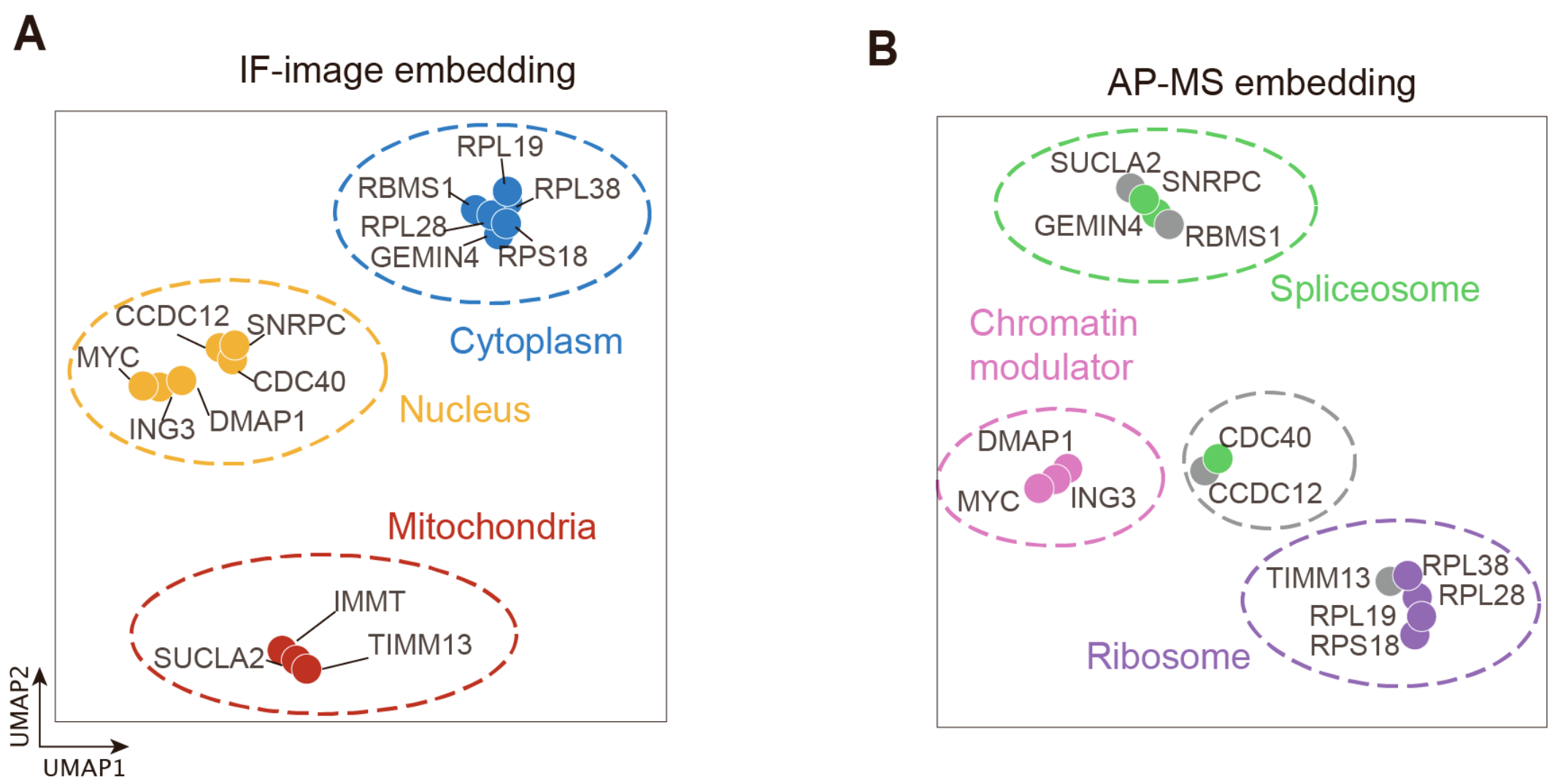

Figure S8. UMAP plot of IF and AP-MS embedding.
a) IF embedding UMAP clusters are annotated with their known subcellular localizations. b) AP-MS embedding UMAP clusters are labeled based on the members within a known complex, the gray dots mean the protein is not known to be part of the complex annotated.

A

Sample #	Tagged cell generation				
	Plasmid library	gRNA cell library	Tagged cell libraries	Clonal cell isolation and validation	Total
100	662	238	336	2,976	4,213
500	2,112	249	387	12,810	15,558
1,000	2,309	272	738	25,404	28,723
5,000	3,336	546	3,690	126,362	133,934
10,000	4,321	890	7,380	252,524	265,115

B

Sample #	Conventional method		HIT-MAP		
	Direct antibody	Total	Tagged cell generation	Anti-tag antibody	Total
100	30,000	30,000	4,213	300	4,513
500	150,000	150,000	15,558	1,200	17,058
1,000	300,000	300,000	28,723	2,400	31,723
5,000	1,500,000	1,500,000	133,934	12,000	148,934
10,000	3,000,000	3,000,000	265,115	24,000	295,115

C

Sample #	Conventional method				HIT-MAP		
	CDS synthesis	Cloning and lentivirus production	Cell culture	Total	Tagged cell generation	Cell culture	Total
100	10,800	5,200	1,575	17,575	4,213	6,300	10,513
500	54,000	26,000	7,875	87,875	15,558	31,500	47,058
1,000	108,000	52,000	15,750	175,750	28,723	63,000	91,723
5,000	540,000	260,000	78,750	878,750	133,934	315,000	448,934
10,000	1,080,000	520,000	157,500	1,757,500	265,115	630,000	895,115

Figure S9. Cost analysis of HITAG-based clonal cell line generation and downstream HIT-MAP applications.

a) Cost breakdown for generating isolated clonal cell lines using HITAG, including gRNA plasmid library construction, integration of gRNAs into cells, tagged cell library construction, and clonal cell isolation and validation. b, c) Cost comparison between conventional methods and HIT-MAP for imaging and AP-MS. For fair comparison, HIT-MAP costs include the tagged cell generation expense, which can be shared across multiple applications for greater cost efficiency but is added at full cost to both IF and AP-MS tallies listed above in case future groups are only interested in using the tagged lines for a single experimental output. The cost comparison evaluates material costs that differ between HIT-MAP and conventional methods during sample preparation and experimental execution. The conventional method costs for imaging were calculated based on the target-specific primary antibodies while AP-MS costs included CDS synthesis, cloning, and transfection/transduction. Detailed costs and parameters are provided in Supplemental Table 16-18. All costs are shown in USD.
